# Supplementary material for: SPOROCYTELESS/NOZZLE cooperates with MADS-domain transcription factors to regulate an auxin-dependent network controlling Megaspore-Mother-Cell differentiation
Source: Nat Commun. 2025 Dec 14;17:683. doi: 10.1038/s41467-025-67343-x (PMC12820212; doi:10.1038/s41467-025-67343-x)
Supplement: Supplementary file 1 — Supplementary Information [file 41467_2025_67343_MOESM1_ESM.pdf]

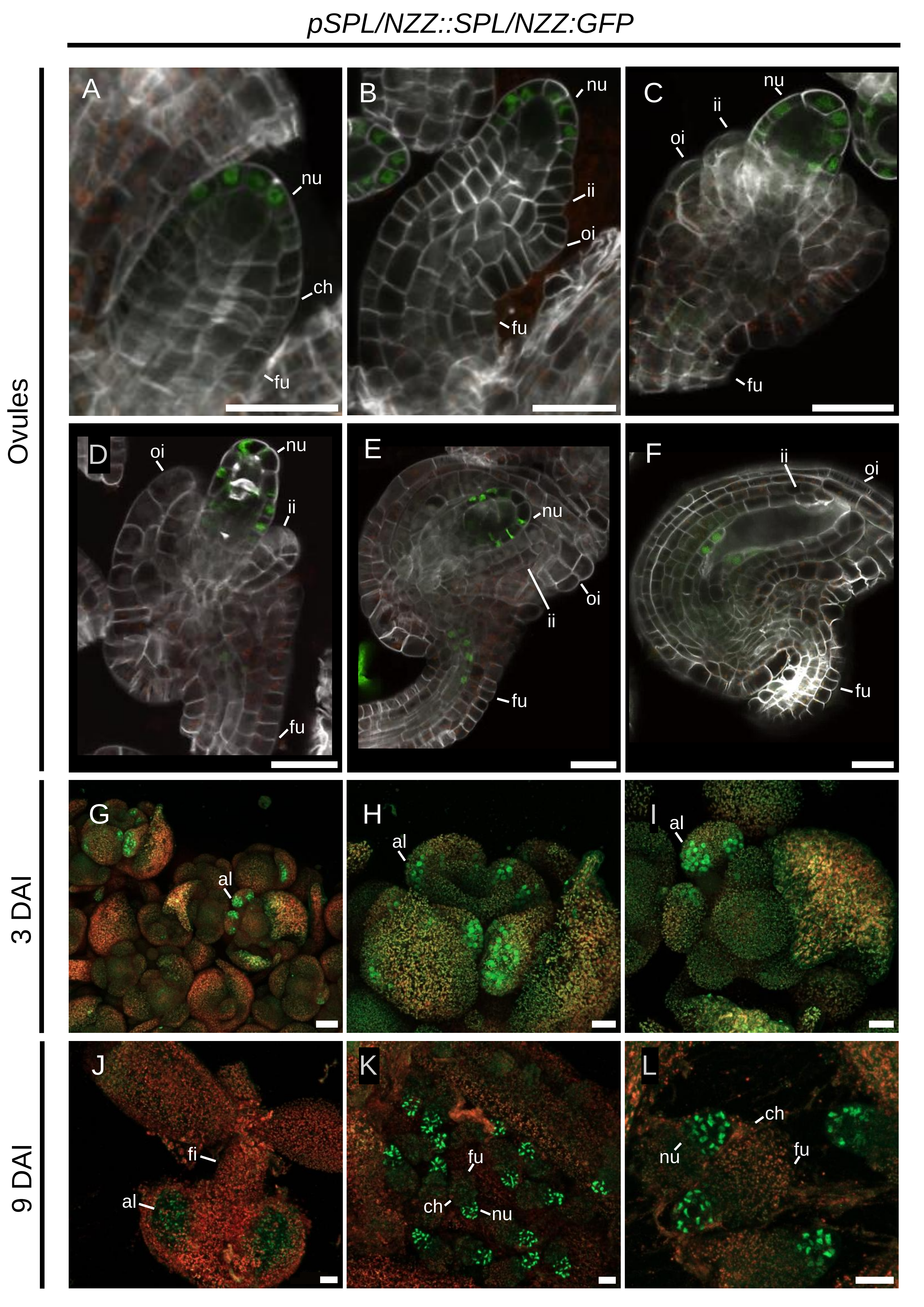

pSPL/NZZ::SPL/NZZ:GFP
Ovules
3 DAI
9 DAI
A
nu
ch
fu
B
nu
ii
oi
fu
C
nu
ii
oi
fu
D
oi
nu
ii
fu
E
nu
oi
ii
fu
F
ii
oi
fu
G
al
H
al
I
al
J
fi
al
K
fu
ch
nu
L
ch
nu
fu

Supplementary Figure 1: *pSPL/NZZ::SPL/NZZ:GFP* expression. (**A-F**) *pSPL/NZZ::SPL/NZZ:GFP* expression during different stages of ovule development. SPL/NZZ is localised in the apical L1 cells at stages 1-II (**A**) and 2-II (**B**). At stage 2-IV (**C**) and 2-V (**D**), SPL/NZZ accumulates in whole nucellar L1 layer. At stage 3-I (**E**), SPL/NZZ is present in the L1 layer and in the central part of the funiculus. At ovule maturity (**F**), the L1 layer degenerates and SPL/NZZ accumulates in the remaining cells. (**G-I**) *pSPL/NZZ::SPL/NZZ:GFP* expression in *pAP1::AP1:GR ap1cal* inflorescences at 3 DAI. At 3 DAI, SPL/NZZ-GFP signal is visible only in developing anthers. (**J-L**) *pSPL/NZZ:SPL/NZZ:GFP* expression in *pAP1::AP1:GR ap1cal* inflorescences at 9 DAI. At 9 DAI, SPL/NZZ-GFP is not visible in anthers anymore (**J**). By contrast, SPL/NZZ-GFP strongly accumulates in ovules nucellus (**K, L**). Abbreviations: nu= nucellus; ch= chalaza; fu= funiculus; ii= inner integument; oi= outer integument; al= anther lobe; fi= filament. (**A-F, H-L**) Scale bar = 20 μm. (**G**) Scale bar = 50 μm.

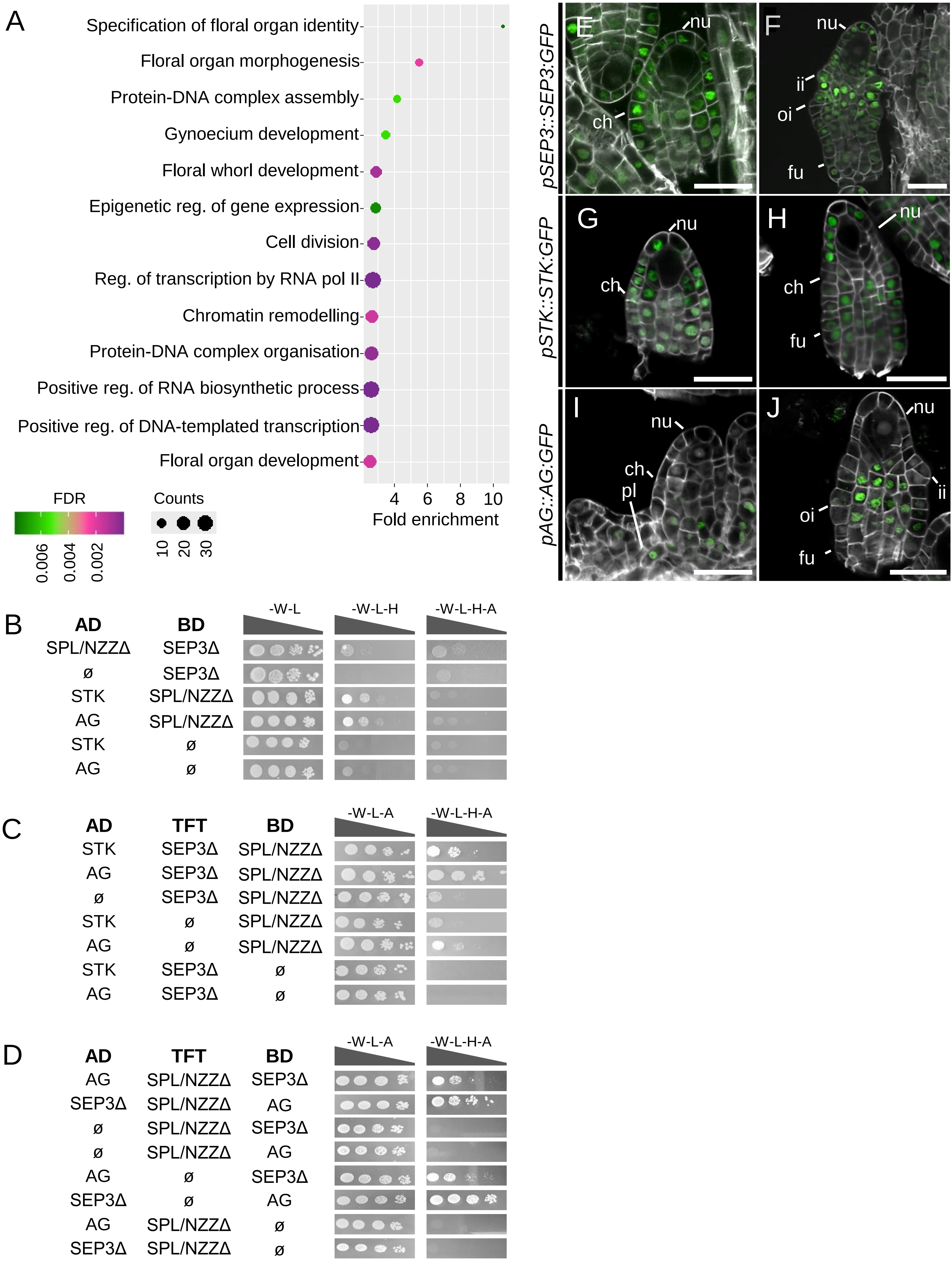
A
Specification of floral organ identity
Floral organ morphogenesis
Protein-DNA complex assembly
Gynoecium development
Floral whorl development
Epigenetic reg. of gene expression
Cell division
Reg. of transcription by RNA pol II
Chromatin remodelling
Protein-DNA complex organisation
Positive reg. of RNA biosynthetic process
Positive reg. of DNA-templated transcription
Floral organ development
FDR
0.006 0.004 0.002
Counts
10 20 30
4 6 8 10
Fold enrichment
pSEP3::SEP3:GFP
pSTK::STK:GFP
pAG::AG:GFP
E F G H I J
nu ch ii oi fu pl
B
AD BD
-W-L -W-L-H -W-L-H-A
SPL/NZZΔ SEP3Δ
ø SEP3Δ
STK SPL/NZZΔ
AG SPL/NZZΔ
STK ø
AG ø
C
AD TFT BD
-W-L-A -W-L-H-A
STK SEP3Δ SPL/NZZΔ
AG SEP3Δ SPL/NZZΔ
ø SEP3Δ SPL/NZZΔ
STK ø SPL/NZZΔ
AG ø SPL/NZZΔ
STK SEP3Δ ø
AG SEP3Δ ø
D
AD TFT BD
-W-L-A -W-L-H-A
AG SPL/NZZΔ SEP3Δ
SEP3Δ SPL/NZZΔ AG
ø SPL/NZZΔ SEP3Δ
ø SPL/NZZΔ AG
AG ø SEP3Δ
SEP3Δ ø AG
AG SPL/NZZΔ ø
SEP3Δ SPL/NZZΔ ø

Supplementary Figure 2: GO analysis of SPL/NZZ interactors identified by the Co-IP/MS, results of yeast-two- and three-hybrid assays and SEP3, STK and AG accumulation patterns in the ovule. (**A**) Biological process GO terms enrichment analysis of the SPL/NZZ putative direct interactors identified by Co-IP/MS. Enriched Go terms with an FDR ≤ 0.05 were considered significant. The complete list of enriched Go terms can be found in Supplementary Data 1. (**B**) Yeast-two-hybrid assay showing interaction between SPL/NZZ and MADS-domain TFs (SEP3, STK, AG). The controls are shown in Figure 1C. Interactions are tested on media depleted of histidine (-W -L -H), or histidine and adenine (-W -L -H -A). For each interaction on the different media, yeast has been spotted at four different concentrations according to a serial dilution (OD 0.5; 1:10; 1:100; 1:1000). (**C, D**) Yeast-three-hybrid assay showing the formation of SPL/NZZ, STK and SEP3 complex, by fusing SPL/NZZ either to the GAL4 BD (**C**) or to the TFT (**D**)**.** Interactions are tested on a medium depleted of histidine (-W -L -H -A). For each interaction, yeast has been spotted at four different concentrations according to a serial dilution (OD 0.5; 1:10; 1:100; 1:1000). (**E, F**) *pSEP3::SEP3:GFP* reporter line showing SEP3 accumulation in ovules at stage 2-I (**E**) and 2-III (**F**). (**G, H**) *pSTK::STK:GFP* reporter line in ovules at stage 2-I (**G**) and 2-II (**H**)**.** (**I, J**) *pAG::AG:GFP* reporter line in ovules at stage 2-I (**I**) and 2-III (**J**)**.** Abbreviations: nu= nucellus; ch= chalaza; fu= funiculus; ii= inner integument; oi= outer integument; pl= placenta. Scale bar = 20 μm

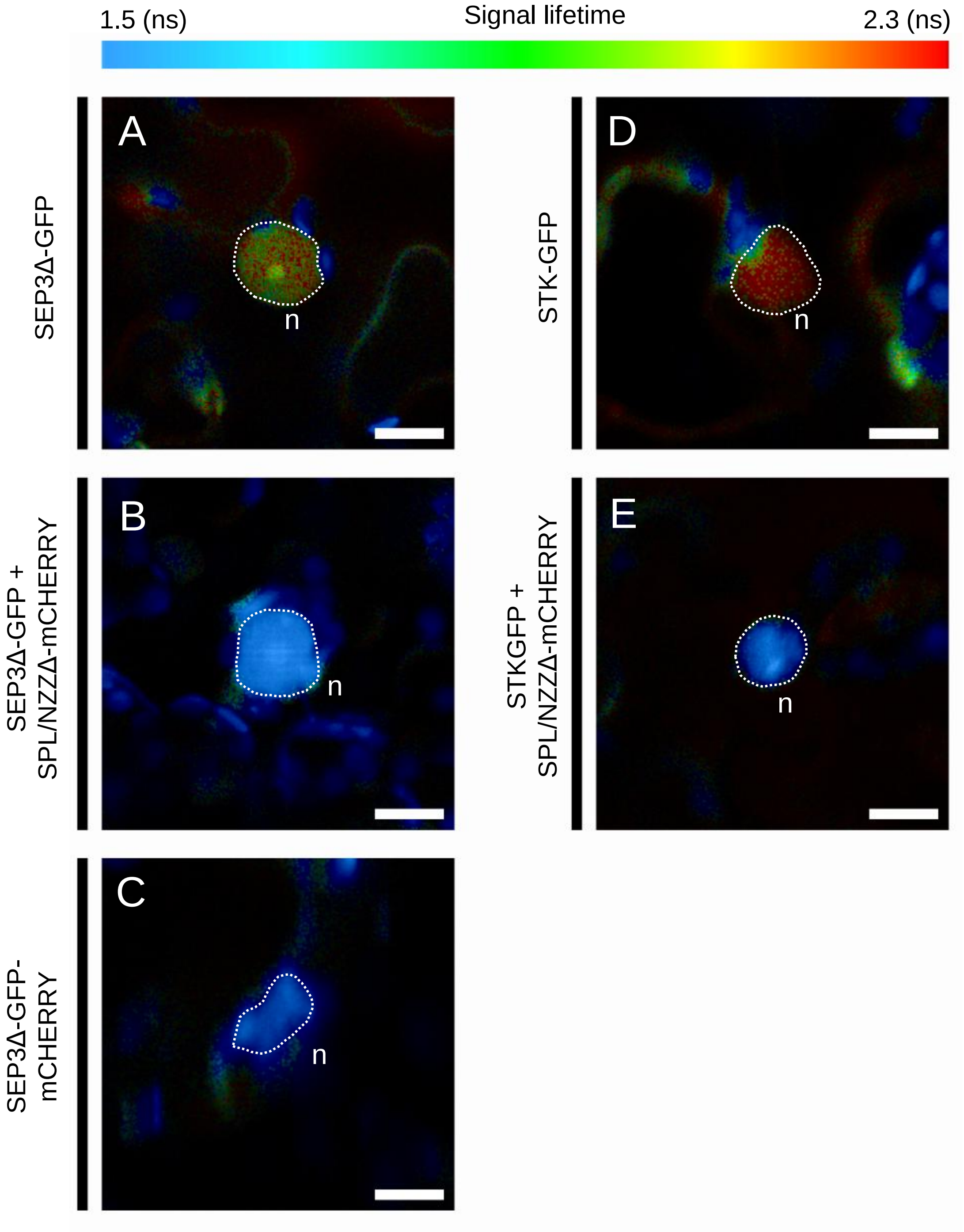

Supplementary Figure 3: Visualisation of the nuclei presented in Figure 1D-H, according to their fluorescence lifetime. (**A**-**E**) The GFP signal imaged from nuclei expressing SEP3Δ-GFP or STK-GFP fusion proteins, either alone (**A**, **D**) or together with the SPL/NZZΔ-mCHERRY fusion protein (**B**, **E**), is represented according to its Lifetime. The SEP3Δ-GFP-mCHERRY fusion protein (**C**) has been used as a positive control. In this case, FRET between GFP and mCHERRY should occur with maximum efficiency. The images are presented with a colourful LUT representing the fluorescent signal lifetime (spanning from 1.5ns to 2.3ns) as evaluated by the SymPhoTime 64 software. Note that the original multichannel images showing either the GFP or the mCHERRY signals in the different nuclei can be found in Figure 1D-H. Nuclei have been highlighted with a dashed line. Abbreviations: n = nucleus. Scale Bars: 10 µm.

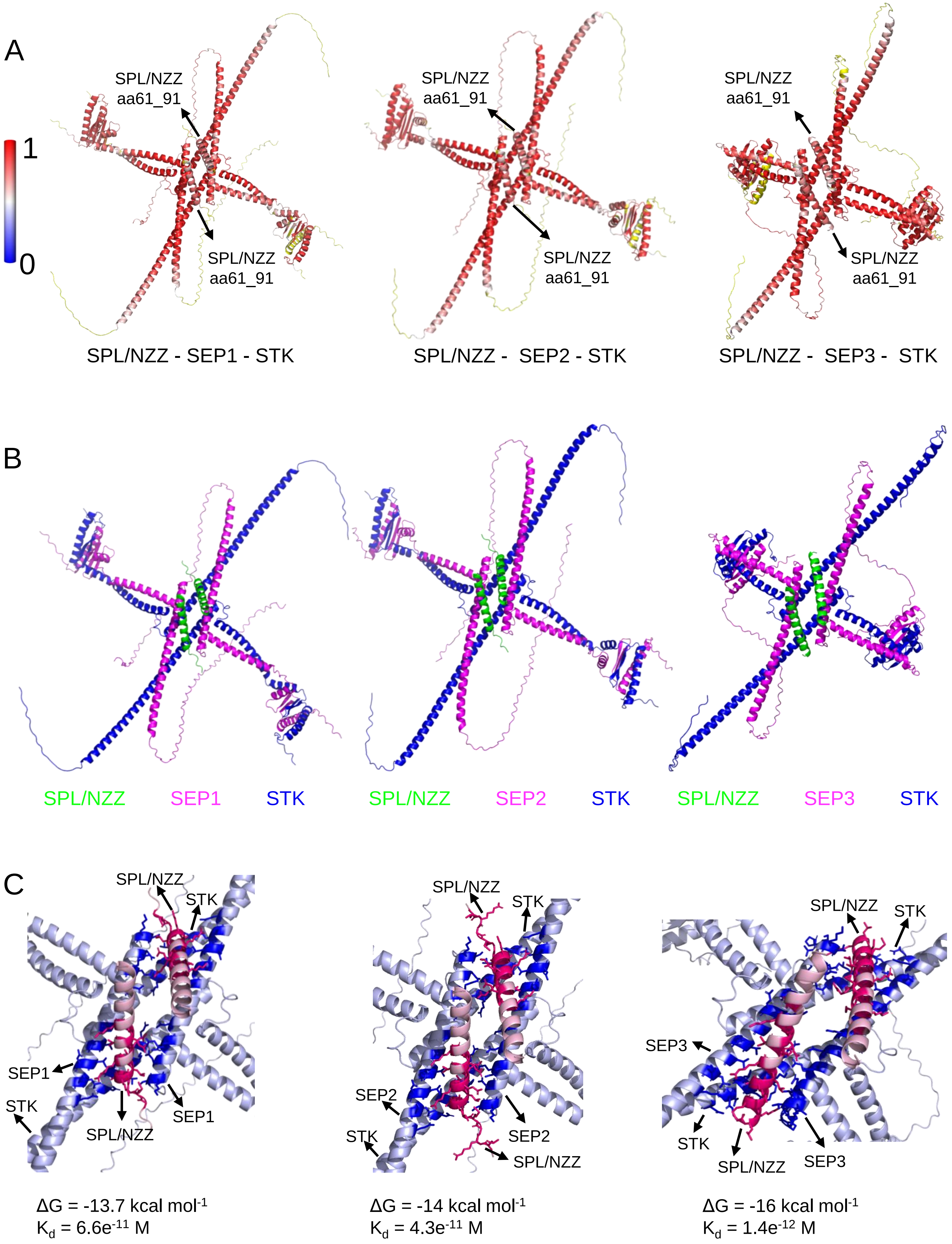
A
1
0
SPL/NZZ aa61_91
SPL/NZZ aa61_91
SPL/NZZ - SEP1 - STK
SPL/NZZ aa61_91
SPL/NZZ aa61_91
SPL/NZZ - SEP2 - STK
SPL/NZZ aa61_91
SPL/NZZ aa61_91
SPL/NZZ - SEP3 - STK
B
SPL/NZZ SEP1 STK
SPL/NZZ SEP2 STK
SPL/NZZ SEP3 STK
C
SPL/NZZ
STK
SEP1
STK
SPL/NZZ
SEP1
SPL/NZZ
STK
SEP2
STK
SEP2
SPL/NZZ
SPL/NZZ
STK
SEP3
STK
SPL/NZZ
SEP3
ΔG = -13.7 kcal mol-1
Kd = 6.6e-11 M
ΔG = -14 kcal mol-1
Kd = 4.3e-11 M
ΔG = -16 kcal mol-1
Kd = 1.4e-12 M

Supplementary Figure 4: AplhaFold3 predictions of SPL/NZZ-SEPs-STK complexes. (**A**) AlphaFold3 predictions of the SPL/NZZ-SEPs-STK complexes (from left to right, SPL/NZZ-SEP1-STK complex, SPL/NZZ-SEP2-STK complex and SPL/NZZ-SEP3-STK complex) coloured according to the prediction confidence. The spectrum bar represents the confidence colours: red colours represent a high confidence (1), while blue colours represent a low confidence (0). (**B**) From left to right, SPL/NZZ-SEP1-STK complex, SPL/NZZ-SEP2-STK complex and SPL/NZZ-SEP3-STK complex. Two copies of STK are shown in blue, two copies of SEP (either 1, 2 or 3) are shown in magenta, and two copies of SPL/NZZ aa61-91 are shown in green. (**C**) Close-up view of the SPL/NZZ-SEPs-STK complex according to a binding-affinity prediction. From left to right, SPL/NZZ-SEP1-STK complex, SPL/NZZ-SEP2-STK complex and SPL/NZZ-SEP3-STK complex. The SPL/NZZ-SEP1-STK complex contains 88 intermolecular contacts between SPL/NZZ and STK-SEP1 at a maximum distance of 5.5 Å. The Gibbs free energy (ΔG) for this complex is -13.7 kcal mol^{-1}, and the dissociation constant (K_d) is 6.6 e^{-11} M. The SPL/NZZ-SEP2-STK complex contains 98 intermolecular contacts between SPL/NZZ and STK-SEP2 at a maximum distance of 5.5 Å. The Gibbs free energy (ΔG) for this complex is -14.0 kcal mol^{-1}, and the dissociation constant (K_d) is 4.3 e^{-11} M. The SPL/NZZ-SEP3-STK complex contains 121 intermolecular contacts between SPL/NZZ and STK-SEP3 at a maximum distance of 5.5 Å. The Gibbs free energy (ΔG) for this complex is -16.0 kcal mol^{-1}, and the dissociation constant (K_d) is 1.4 e^{-12} M. For all complexes, STK and SEPs amino acids that take contact with SPL/NZZ are shown in blue. SPL/NZZ amino acids that participate in binding to STK and SEPs are depicted in red. Light blue and light red colouring marks regions where there is no binding. It is possible to notice that one SPL/NZZ molecule interacts with the K-domain of one STK molecule and two SEPs molecules.

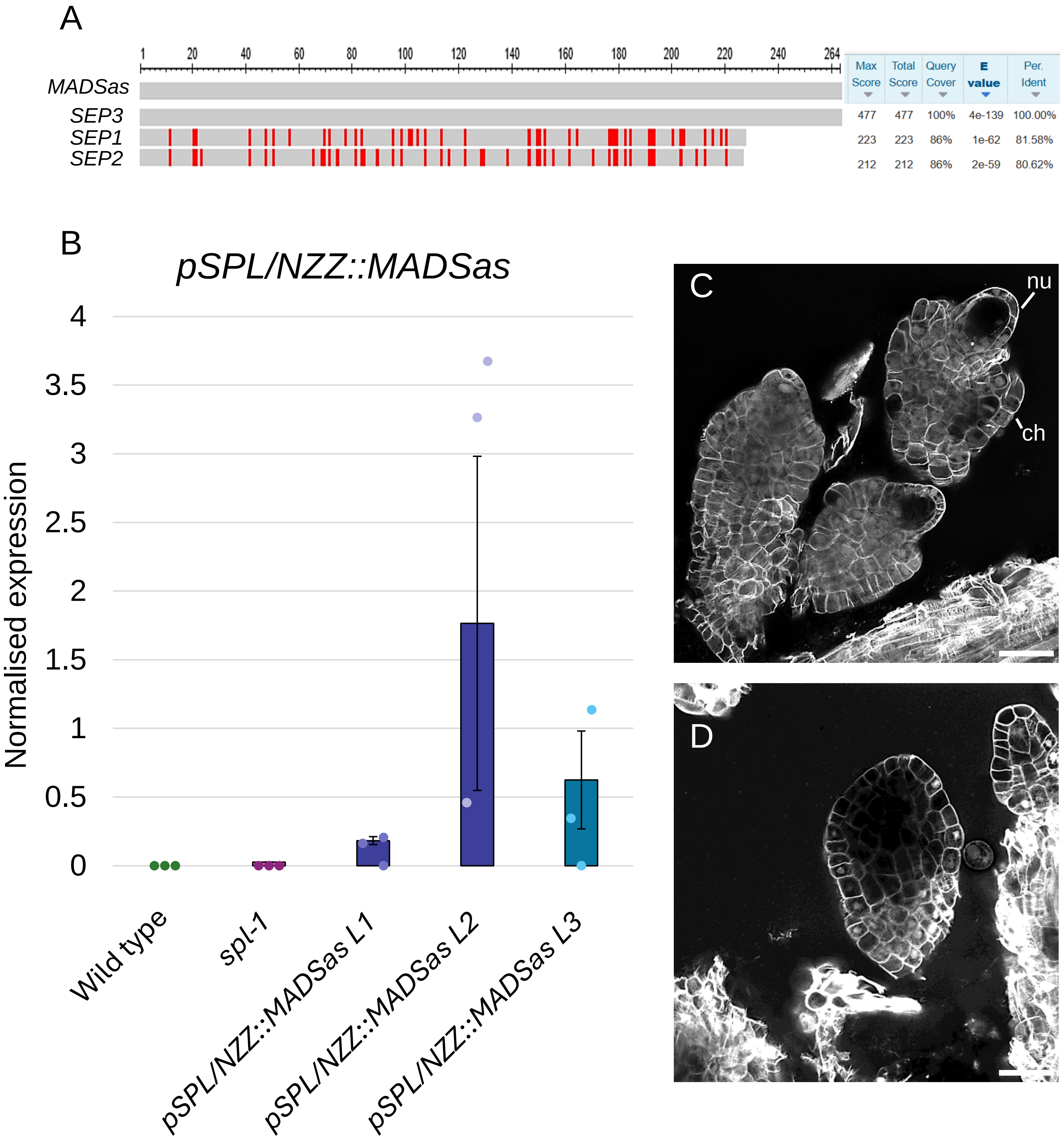
A
1 20 40 60 80 100 120 140 160 180 200 220 240 264
MADSas
SEP3
SEP1
SEP2
Max Score
Total Score
Query Cover
E value
Per. Ident
477 477 100% 4e-139 100.00%
223 223 86% 1e-62 81.58%
212 212 86% 2e-59 80.62%
B
pSPL/NZZ::MADSas
Normalised expression
4
3.5
3
2.5
2
1.5
1
0.5
0
Wild type
spl-1
pSPL/NZZ::MADSas L1
pSPL/NZZ::MADSas L2
pSPL/NZZ::MADSas L3
C
nu
ch
D

Supplementary Figure 5: *MADSas* sequence homology to *SEP1, 2* and *3* and *MADSas* expression and ovule phenotypes in *pSPL/NZZ::MADSas* lines. (**A**) Multiple sequence alignment among *MADSas, SEP1, SEP2* and *SEP3* transcripts performed with the NCBI BLASTn tool. The *MADSas* is transcribed from a 264bp portion amplified from *SEP3 MADS* box sequence and placed in reverse under the control of the *SPL/NZZ* promoter. The *MADSas* has around 80% sequence homology with *SEP1* and *2.* In the schematic of the alignment, mismatched bases are depicted in red. (**B**) Bar plot showing *pSPL/NZZ::MADSas* normalised expression in inflorescences from wild type, *spl-1* and the three independent *pSPL/NZZ::MADSas* lines. *ACTIN8* was used as the housekeeping gene. Bars represent the mean ± SEM of the expression, as evaluated from three technical replicates per each sample. Dots within the bars represent individual values for each technical replicate. Source data are provided as a Source Data file. Primers used are listed in Supplementary Data 4. (**C**, **D**) Examples of ovule phenotypes observed in *pSPL/NZZ::MADSas* lines. While a portion of the ovule did not develop the MMC (**C**), the structure of few ovules appears severely impaired, hindering the identification of the different ovule domains (**D**). Such ovules (**D**) were not considered for the evaluation of the MMC differentiation and were excluded from the analysis. As reported in Figure 2, the analysis of ovules developing an MMC has been performed on 5, 5, 5, 9 and 4 different pistils, respectively, for the wild type (122 ovules observed in total), *spl-1* (110 ovules observed in total), *pSPL/NZZ::MADSas L1* (113 ovules observed in total), *pSPL/NZZ::MADSas L2* (208 ovules observed in total), *pSPL/NZZ::MADSas L3* (67 ovules observed in total). The percentage of ovules developing an MMC is reported in Figure 2F. Source data are provided as a Source Data file. Abbreviations: nu= nucellus; ch= chalaza; MMC= megaspore mother cell. Scale bar = 20 µm.

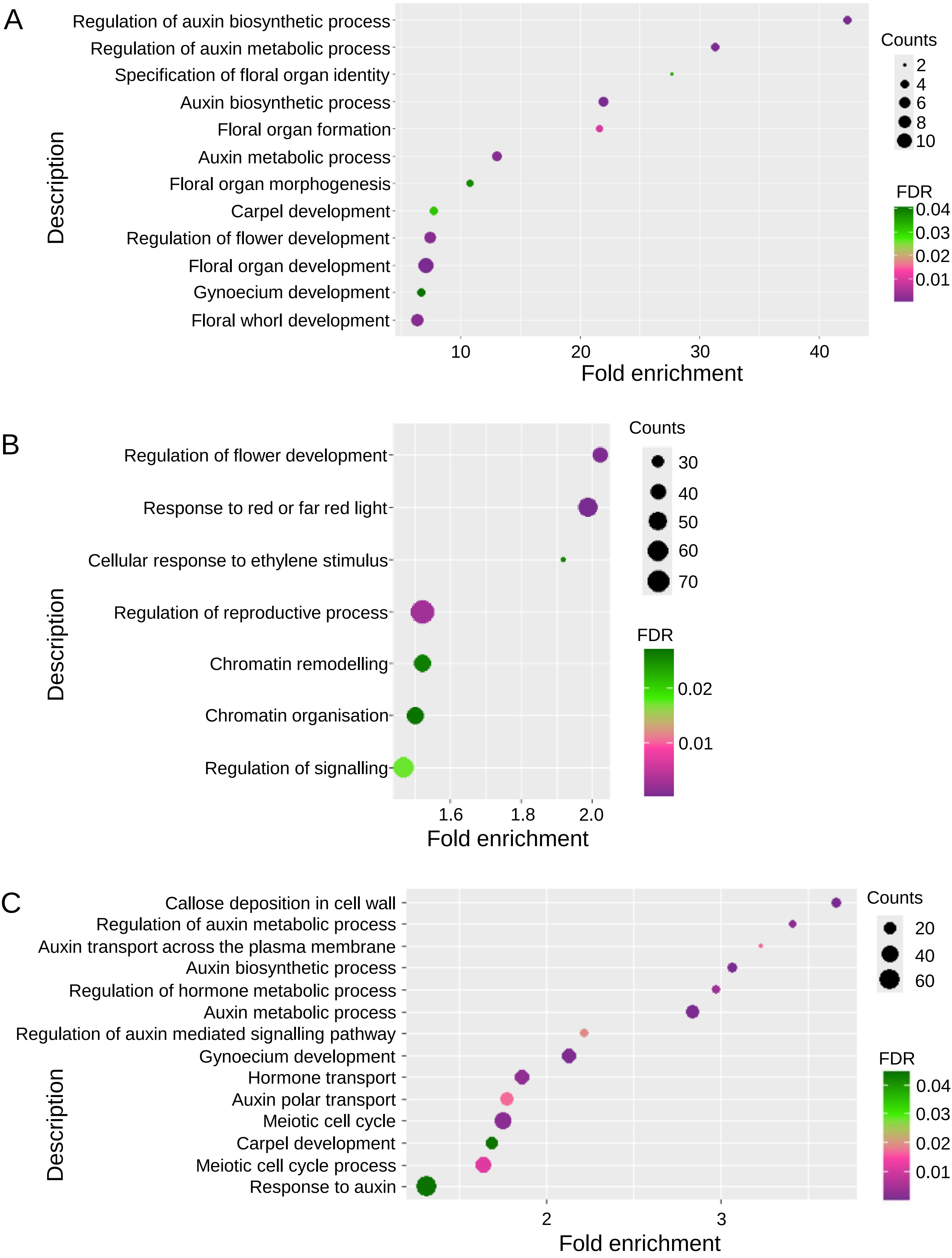
A
Regulation of auxin biosynthetic process
Regulation of auxin metabolic process
Specification of floral organ identity
Auxin biosynthetic process
Floral organ formation
Auxin metabolic process
Floral organ morphogenesis
Carpel development
Regulation of flower development
Floral organ development
Gynoecium development
Floral whorl development
Description
10
20
30
40
Fold enrichment
Counts
2
4
6
8
10
FDR
0.04
0.03
0.02
0.01
B
Regulation of flower development
Response to red or far red light
Cellular response to ethylene stimulus
Regulation of reproductive process
Chromatin remodelling
Chromatin organisation
Regulation of signalling
Description
1.6
1.8
2.0
Fold enrichment
Counts
30
40
50
60
70
FDR
0.02
0.01
C
Callose deposition in cell wall
Regulation of auxin metabolic process
Auxin transport across the plasma membrane
Auxin biosynthetic process
Regulation of hormone metabolic process
Auxin metabolic process
Regulation of auxin mediated signalling pathway
Gynoecium development
Hormone transport
Auxin polar transport
Meiotic cell cycle
Carpel development
Meiotic cell cycle process
Response to auxin
Description
2
3
Fold enrichment
Counts
20
40
60
FDR
0.04
0.03
0.02
0.01

Supplementary Figure 6: Biological processes GO terms enrichment analysis of SPL/NZZ and SEP3 common target genes and of DE genes in the *spl-1* pistil with respect to the wild type. (**A**) Biological process GO term enrichment analysis, done on the 182 genes that have overlapping peaks between SPL/NZZ and SEP3 ChIPseq experiments. Categories associated with auxin and hormone processes, flower and floral organs development are enriched. Enriched GO terms with an FDR ≤ 0.05 were considered significant. The complete list of enriched GO terms can be found in Supplementary Data 2. (**B**) Biological process GO terms enrichment analysis conducted on the downregulated genes in *spl-1* pistils with respect to wild type. Categories associated with flower development and chromatin organisation are among the most enriched categories. The complete list of enriched GO terms can be found in Supplementary Data 3. (**C**) Biological process GO terms enrichment analysis conducted on the upregulated genes in *spl-1* pistils with respect to wild type. Categories associated with meiosis, gynoecium development, auxin and hormone processes are among the most enriched categories. The complete list of enriched GO terms can be found in Supplementary Data 3.

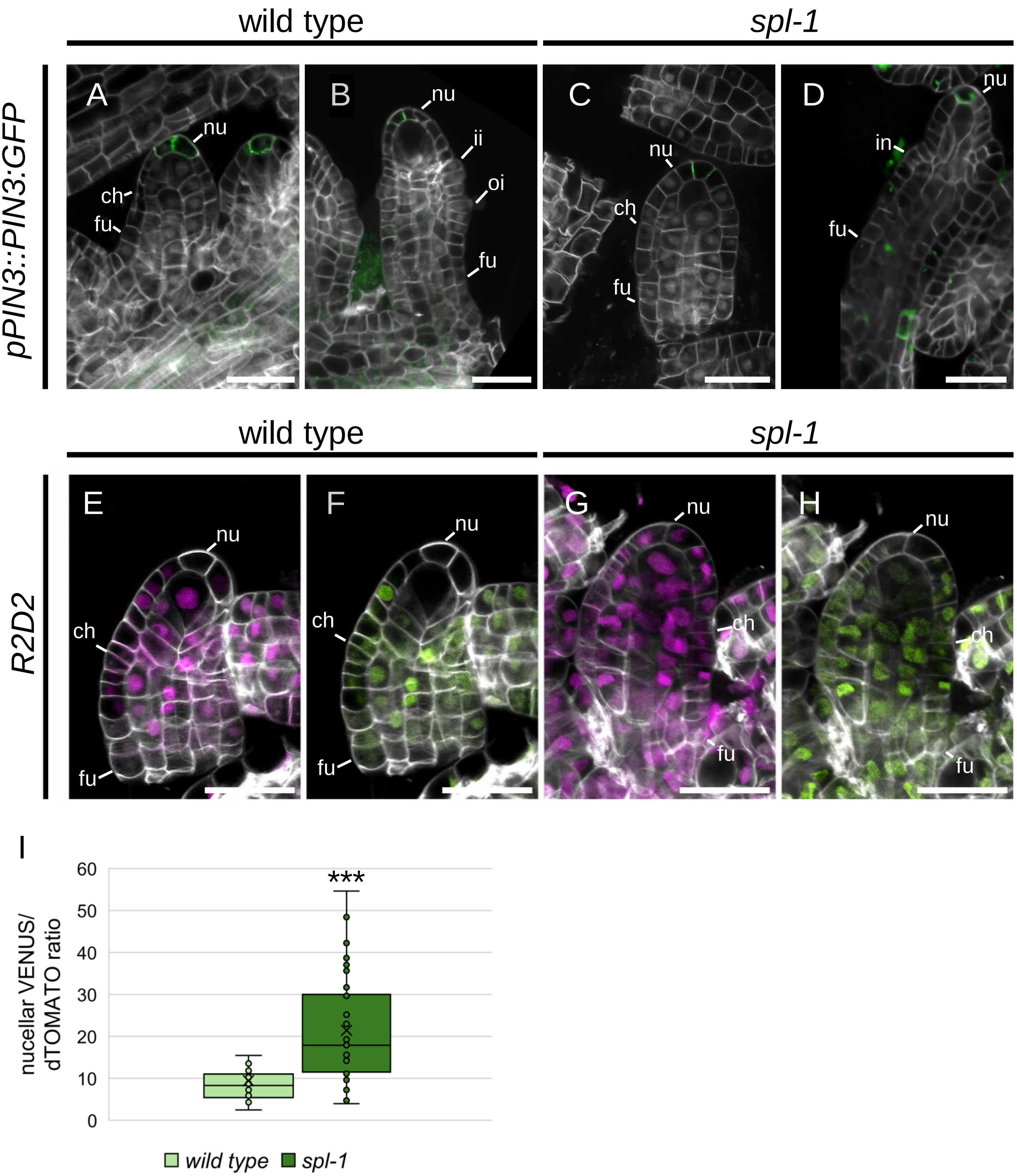

wild type
spl-1
pPIN3::PIN3:GFP
A
B
C
D
nu
ch
fu
ii
oi
in
R2D2
E
F
G
H
I
60
50
40
30
20
10
0
***
nucellar VENUS/
dTOMATO ratio
wild type
spl-1

Supplementary Figure 7: *pPIN3::PIN3:GFP* expression in wild type and *spl-1* ovules and *R2D2* signal in wild type and *spl-1* ovules at stage 1-II. (**A, B**) PIN3-GFP accumulation at stage 2-I (**A**) and 2-II (**B**) in wild type. (**C, D**) PIN3-GFP accumulation at stage 2-I (**C**) and 2-II (**D**) in *spl-1*. (**E-H**) *R2D2* reporter line in wild type (**E**, **F**) and *spl-1* (**G**, **H**) ovules at stage 2-I, showing mDII-tdTOMATO (**E**, **G**) and DII-VENUS (**F**, **H**) accumulation. (**I**) Box plot showing the tdTOMATO/VENUS signal ratio from the *R2D2* reporter, in the wild type and *spl-1* nucella at stage 2-III. The analysis has been performed on 28 and 45 nuclei, from 3 wild type and 4 *spl-1* different ovules, respectively. Source data are provided as a Source Data file. Asterisks over boxes represent the statistical significance as determined by student's t-test, two-sided distribution, homoscedastic, confronting the mutant with the wild-type condition. *** = p < 0.001. Exact P values for each comparison can be found in the Source Data file. Box-plots elements correspond to: centre line = median; X = box limits = interquartile range; whiskers = lowest and highest values in the 1.5 interquartile range. Single measures are represented with dots in the boxes. Abbreviations: nu= nucellus; ch= chalaza; fu= funiculus; ii= inner integument; oi= outer integument; in= integument.
Scale bar = 20 µm.

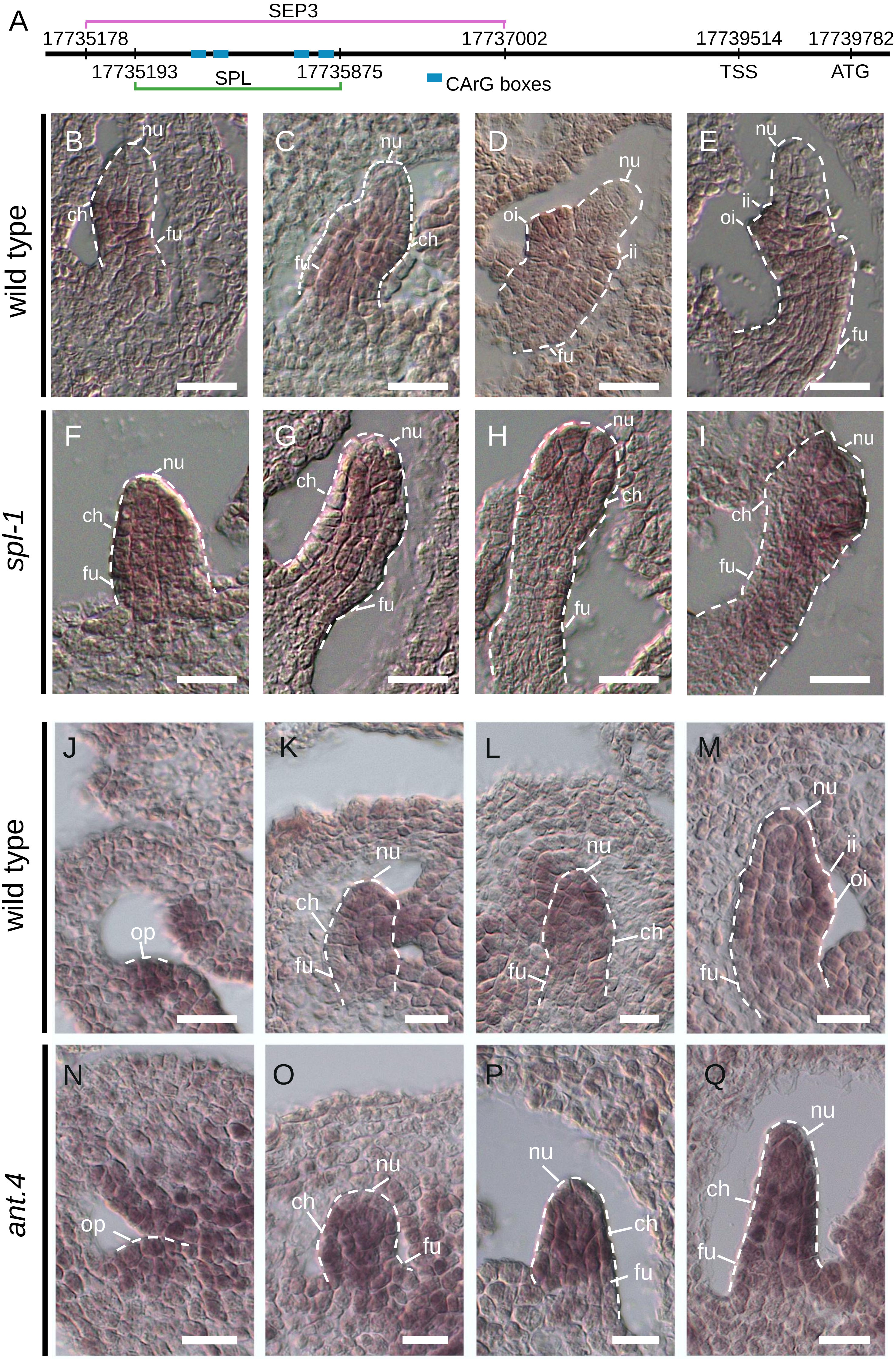
A
SEP3
17735178
17737002
17739514
17739782
17735193
SPL
17735875
CArG boxes
TSS
ATG
wild type
B
C
D
E
nu
ch
fu
oi
ii
spl-1
F
G
H
I
wild type
J
K
L
M
op
ant.4
N
O
P
Q

Supplementary Figure 8: SPL/NZZ and SEP3 ChIPseq peaks on *ANT* promoter, *ANT* expression in wild type and *spl-1* ovules and *PIN1* expression in wild type and *ant.4* ovules. (**A**) Schematic of the *ANT* promoter region showing, with a green box, the peak identified in the SPL/NZZ ChIPseq and in magenta the peak identified in the SEP3 ChIPseq. The 4 CArG-box sequences identified by the MEME analysis on the SPL/NZZ peak are highlighted by blue boxes. (**B**-**I**) *ANT* transcript *in-situ* hybridisation on wild type (**B-E**) and *spl-1* (**F-I**) ovules. In wild-type ovules, *ANT* is expressed in the chalaza and the funiculus (**B-E**), whereas *ANT* transcript accumulates ectopically also in the nucellus in the *spl-1* mutant (**F-I**). (**J**-**Q**) *PIN1* expression detected by *in-situ* hybridisation in wild type (**J**-**M**) and *ant.4* (**N**-**Q**) ovules at stages 0-II (**J**, **N**), 1-II (**K**, **O**), 2-I (**L**, **P**) and 2-III (**M**, **Q**). At stages 2-I, *PIN1* expression could be observed mainly in the nucellus of the wild-type ovule (**L**). At stage 2-III (**M**), *PIN1* is still visible in the nucellus and partially in the chalaza. By contrast, in the *ant.4* ovule (**N**-**Q**), *PIN1* expression can be observed in the whole ovule primordia. Abbreviations: op= ovule primordia; nu= nucellus; ch= chalaza; fu= funiculus; ii= inner integument; oi= outer integument. Scale bars = 20µm.

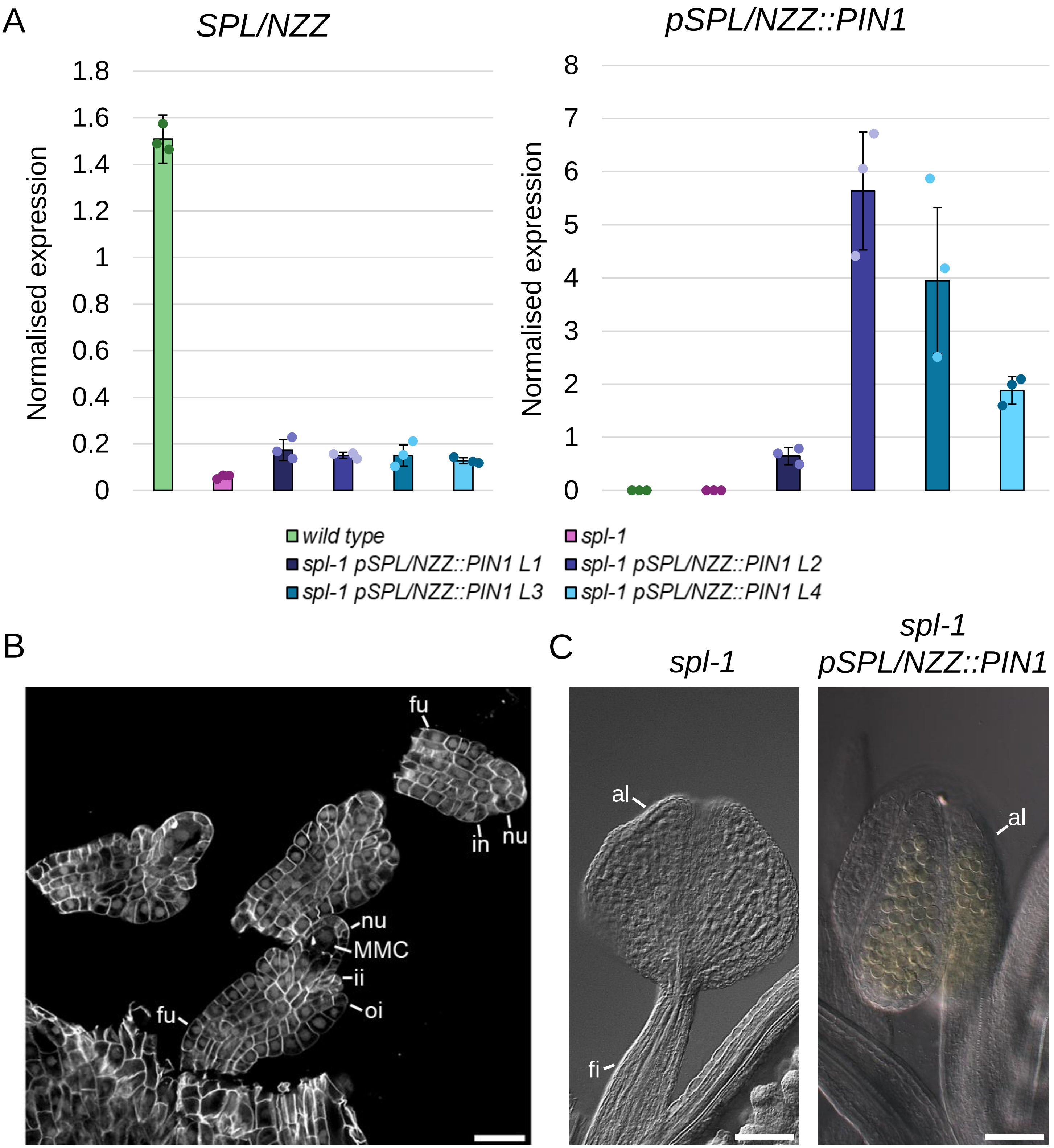
A
SPL/NZZ
Normalised expression
1.8
1.6
1.4
1.2
1
0.8
0.6
0.4
0.2
0
pSPL/NZZ::PIN1
Normalised expression
8
7
6
5
4
3
2
1
0
wild type
spl-1
spl-1 pSPL/NZZ::PIN1 L1
spl-1 pSPL/NZZ::PIN1 L2
spl-1 pSPL/NZZ::PIN1 L3
spl-1 pSPL/NZZ::PIN1 L4
B
fu
in
nu
nu
MMC
ii
oi
fu
C
spl-1
spl-1 pSPL/NZZ::PIN1
al
fi
al

Supplementary Figure 9: *pSPL/NZZ::PIN1* expression and ovule and anther phenotypes in *spl-1 pSPL/NZZ::PIN1* plants. (**A**) Bar plot showing *SPL/NZZ* normalised expression in inflorescences from the wild type, *spl-1* and four independent *spl-1 pSPL/NZZ::PIN1* lines and Bar plot showing *pSPL/NZZ::PIN1* normalised expression in the same samples. *ACTIN8* was used as the housekeeping gene. Bars represent the mean ± SEM of the expression, as evaluated from three technical replicates per each sample. Dots within the bars represent individual values for each technical replicate. Source data are provided as a Source Data file. Primers used are listed in Supplementary Data 4. (**B**) Despite the ability of *pSPL/NZZ::PIN1* to rescue the MMC specification, few ovules still resemble the *spl-1* phenotype. As reported in Figure 4, the analysis of ovules developing an MMC has been performed on 8, 7, 8, 6, 8 and 9 different pistils, respectively, for the wild type (202 ovules observed in total), *spl-1* (238 ovules observed in total), *spl-1 pSPL/NZZ::PIN1 L1* (294 ovules observed in total), *spl-1 pSPL/NZZ::PIN1 L2* (196 ovules observed in total), *spl-1 pSPL/NZZ::PIN1 L3* (274 ovules observed in total) and *spl-1 pSPL/NZZ::PIN1 L4* (364 ovules observed in total). The percentage of ovules developing an MMC is reported in Figure 4P. Source data are provided as a Source Data file. (**C**) *spl-1* and *spl-1 pSPL/NZZ::PIN1* anthers. In contrast to the *spl-1* situation, *spl-1 pSPL/NZZ::PIN1* could develop anthers generating wild-type-like pollen grains. Abbreviations: nu= nucellus; fu= funiculus; ii= inner integument; oi= outer integument; in= integument; MMC= megaspore mother cell; al = anther lobe; fi = anther filament. (**B**) Scale bar = 20 μm. (**C**)
Scale bar = 50 μm.

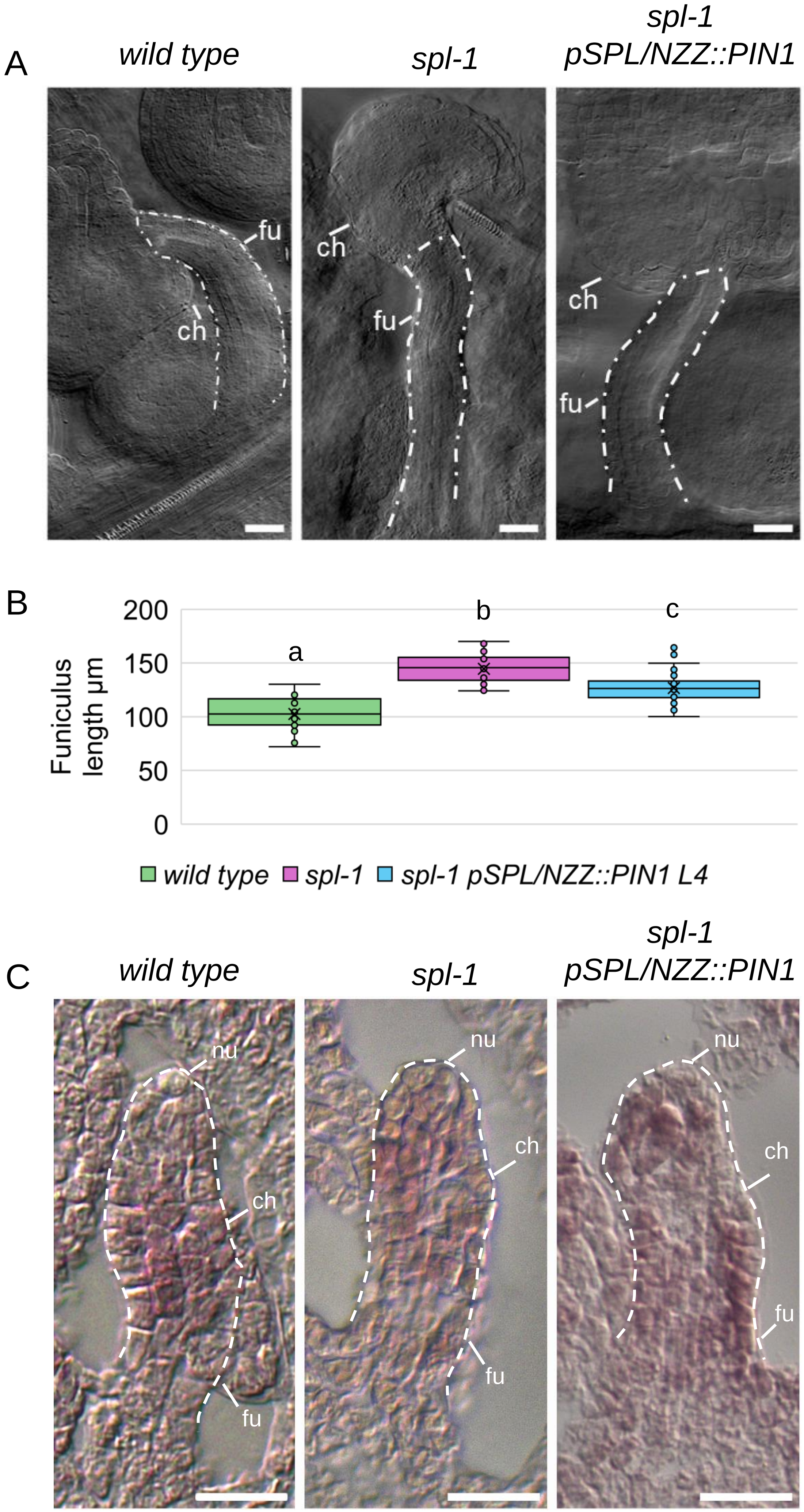

A
wild type
spl-1
spl-1
pSPL/NZZ::PIN1
fu
ch
ch
fu
ch
fu
B
Funiculus
length µm
200
150
100
50
0
a
b
c
wild type
spl-1
spl-1 pSPL/NZZ::PIN1 L4
C
wild type
spl-1
spl-1
pSPL/NZZ::PIN1
nu
ch
fu
nu
ch
fu
nu
ch
fu

Supplementary Figure 10: Funiculus length and *ANT* expression in *spl-1 pSPL/NZZ::PIN1.* (**A**, **B**) Funiculus length in wild type, *spl-1* and *spl-1 pSPL/NZZ::PIN1* ovules at stage 3-VI. Even though the ovule development was restored in *spl-1 pSPL/NZZ::PIN1* (**A**), funiculi were still statistically longer than the wild-type ones. Nevertheless, *spl-1 pSPL/NZZ::PIN1* funiculi resulted statistically shorter than the *spl-1* ones (**B**). Measurements were performed on 25, 33 and 45 different funiculi, respectively, for the wild type, *spl-1* and *spl-1 pSPL/NZZ::PIN1 L4.* Source data are provided as a Source Data file. Letters above the box plots indicate homogenous categories with $p \leq 0.05$, as determined by one-way ANOVA with post-hoc Tukey HSD test. Exact P values for each comparison can be found in the Source Data file. Box-plots elements correspond to: centre line = median; X= average; box limits = interquartile range; whiskers = lowest and highest values in the 1.5 interquartile range. Single measures are represented with dots in the boxes. (**C**) *ANT* transcript *in-situ* hybridisation on wild type, *spl-1* and *spl-1 pSPL/NZZ::PIN1 L4* ovules. Despite the MMC differentiation being restored in *spl-1 pSPL/NZZ::PIN1,* SPL/NZZ direct target as *ANT,* remains ectopically expressed, similarly to the *spl-1* situation. Abbreviations: nu= nucellus; ch= chalaza; fu= funiculus. Scale bars = 20 µm.

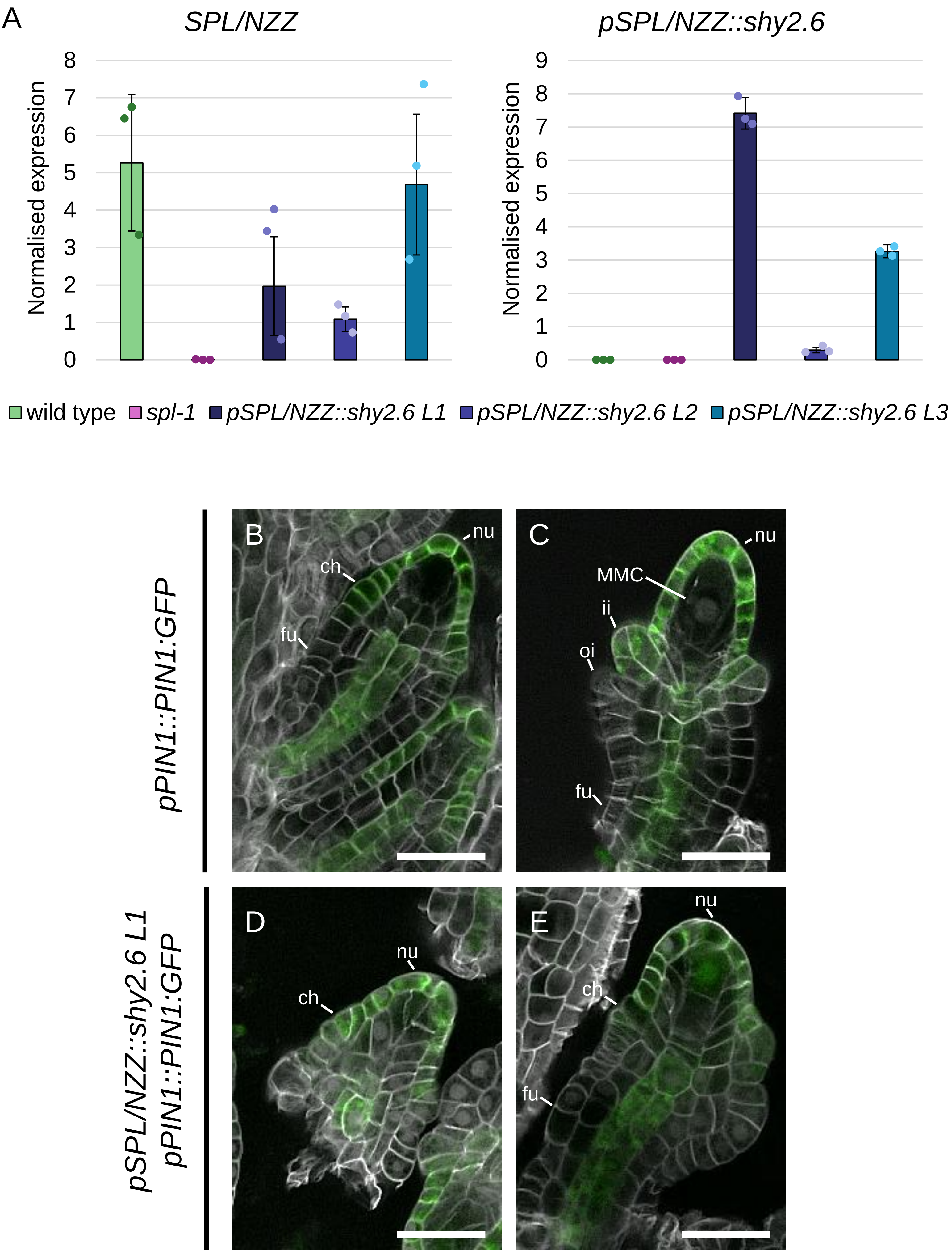

A
SPL/NZZ
Normalised expression
0
1
2
3
4
5
6
7
8
pSPL/NZZ::shy2.6
Normalised expression
0
1
2
3
4
5
6
7
8
9
wild type
spl-1
pSPL/NZZ::shy2.6 L1
pSPL/NZZ::shy2.6 L2
pSPL/NZZ::shy2.6 L3
pPIN1::PIN1:GFP
pSPL/NZZ::shy2.6 L1
pPIN1::PIN1:GFP
B
nu
ch
fu
C
nu
MMC
ii
oi
fu
D
nu
ch
E
nu
ch
fu

Supplementary Figure 11: *pSPL/NZZ::shy2.6* expression level and PIN1-GFP accumulation in *pSPL/NZZ::shy2.6.* (**A**) Bar plot showing *SPL/NZZ* and *pSPL/NZZ::shy2.6* normalised expressions in inflorescences from wild type, *spl-1* and three independent *pSPL/NZZ::shy2.6* lines. *ACTIN8* was used as housekeeping gene for normalisation. Bars represent the mean ± SEM of the expression, as evaluated from three technical replicates per each sample. Dots within the bars represent individual values for each technical replicate. Source data are provided as a Source Data file. Primers used are listed in Supplementary Data 4. (**B**-**E**) PIN1-GFP accumulation in wild type (**B**, **C**) and *pSPL/NZZ::shy2.6 L1* (**D**, **E**) ovules at stages 2-I (**B**, **D**) and 2-III (**C**, **E**). Abbreviations: nu= nucellus; ch= chalaza; fu= funiculus; ii= inner integument; oi= outer integument; MMC= Megaspore mother cell. Scale bar = 20 μm.

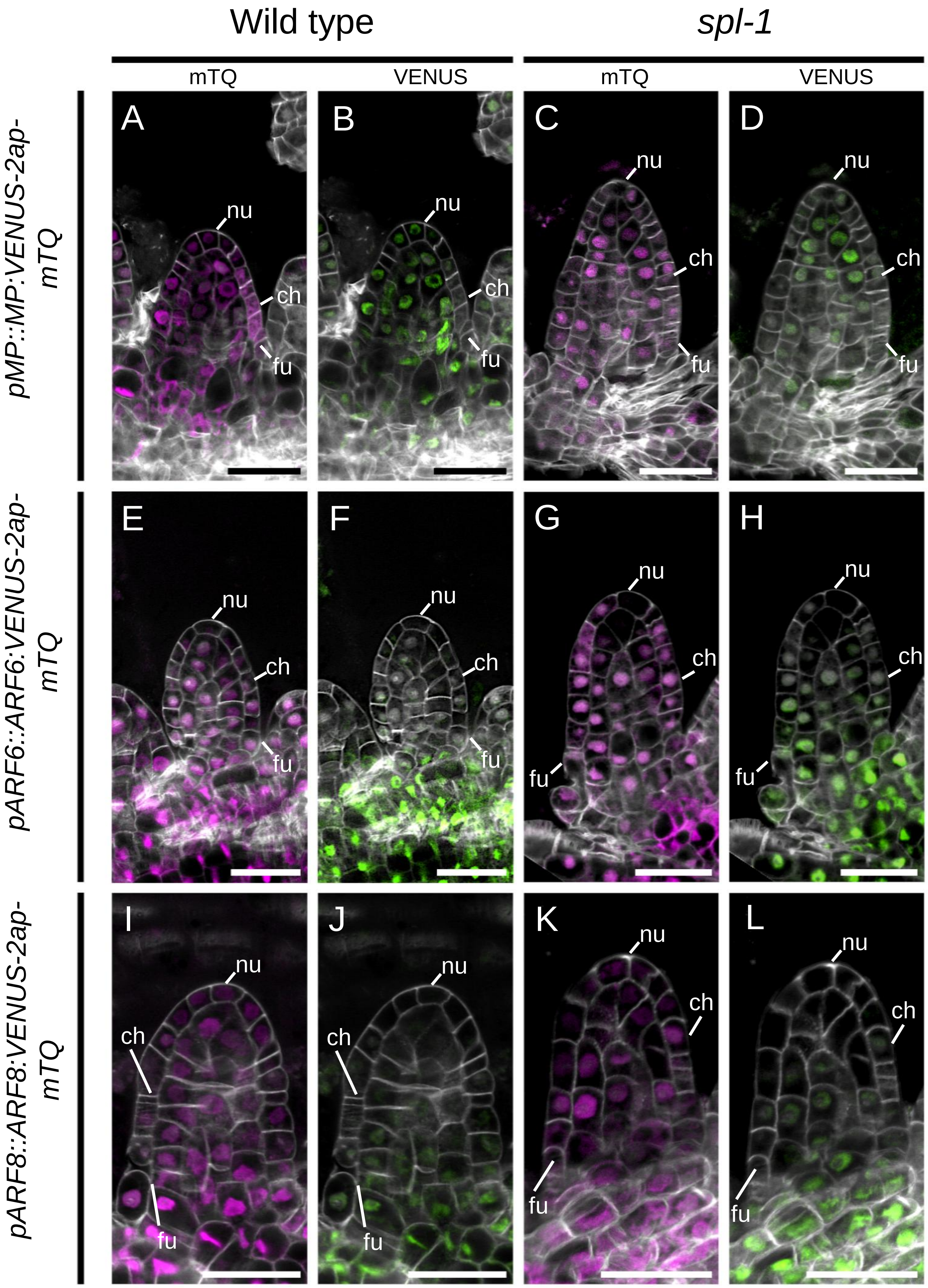
Wild type
spl-1
mTQ
VENUS
mTQ
VENUS
pMP::MP:VENUS-2ap-mTQ
pARF6::ARF6:VENUS-2ap-mTQ
pARF8::ARF8:VENUS-2ap-mTQ
A
B
C
D
E
F
G
H
I
J
K
L
nu
ch
fu

Supplementary Figure 12: ClassA ARFs expression in wild-type and *spl-1* ovules. (**A-D**) *pMP::MP:VENUS-2ap-mTQ* reporter in wild type (**A**, **B**) and *spl-1* (**C**, **D**) ovules. (**E-H**) *pARF6::ARF6:VENUS-2ap-mTQ* reporter in wild type (**E**, **F**) and *spl-1* (**G**, **H**) ovules. (**I-L**) *pARF8::ARF8:VENUS-2ap-mTQ* reporter in wild type (**I**, **J**) and *spl-1* (**K**, **L**) ovules. *MP, ARF6* and *ARF8* domains of expression and translation are visualised thanks to the mTQ signal (**A**, **C**, **E**, **G**, **I**, **K**), while domains of protein accumulation are visualised by the VENUS signal (**B**, **D**, **F**, **H**, **J**, **L**). Abbreviations: nu= nucellus; ch= chalaza; fu= funiculus. Scale bar = 20 μm.

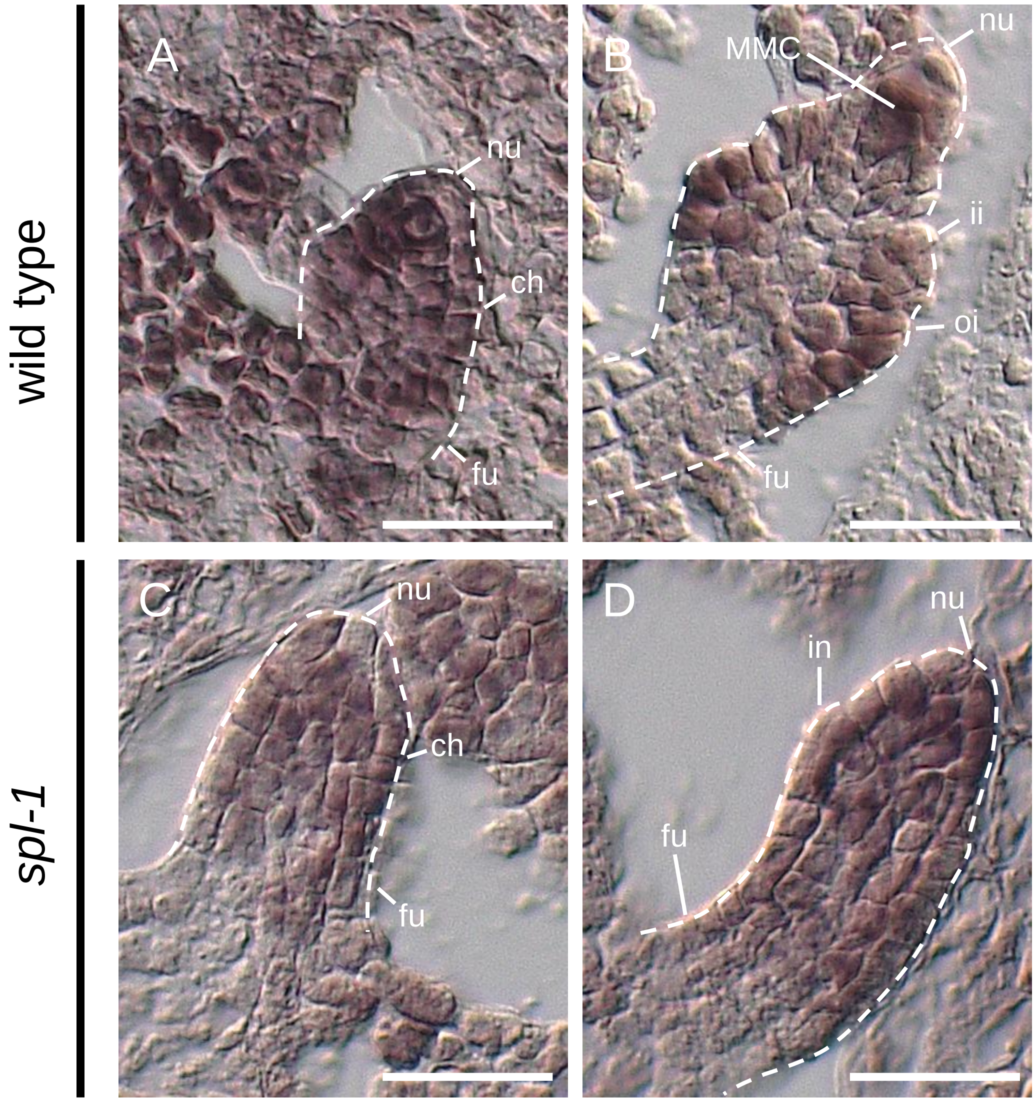

Supplementary Figure 13: *ARF9* expression in wild-type and *spl-1* ovules detected by *in-situ* hybridisation. (**A, D**) ISH showing *ARF9* expression in wild-type ovules (**A**, **B**) and *spl-1* (**C, D**) ovules at stages 1-II (**A, C**) and 2-III (**B, D**). Abbreviations: nu= nucellus; ch= chalaza; fu= funiculus; MMC= megaspore mother cell; ii= inner integument; oi= outer integument; in= integument. Scale bar = 20 μm.

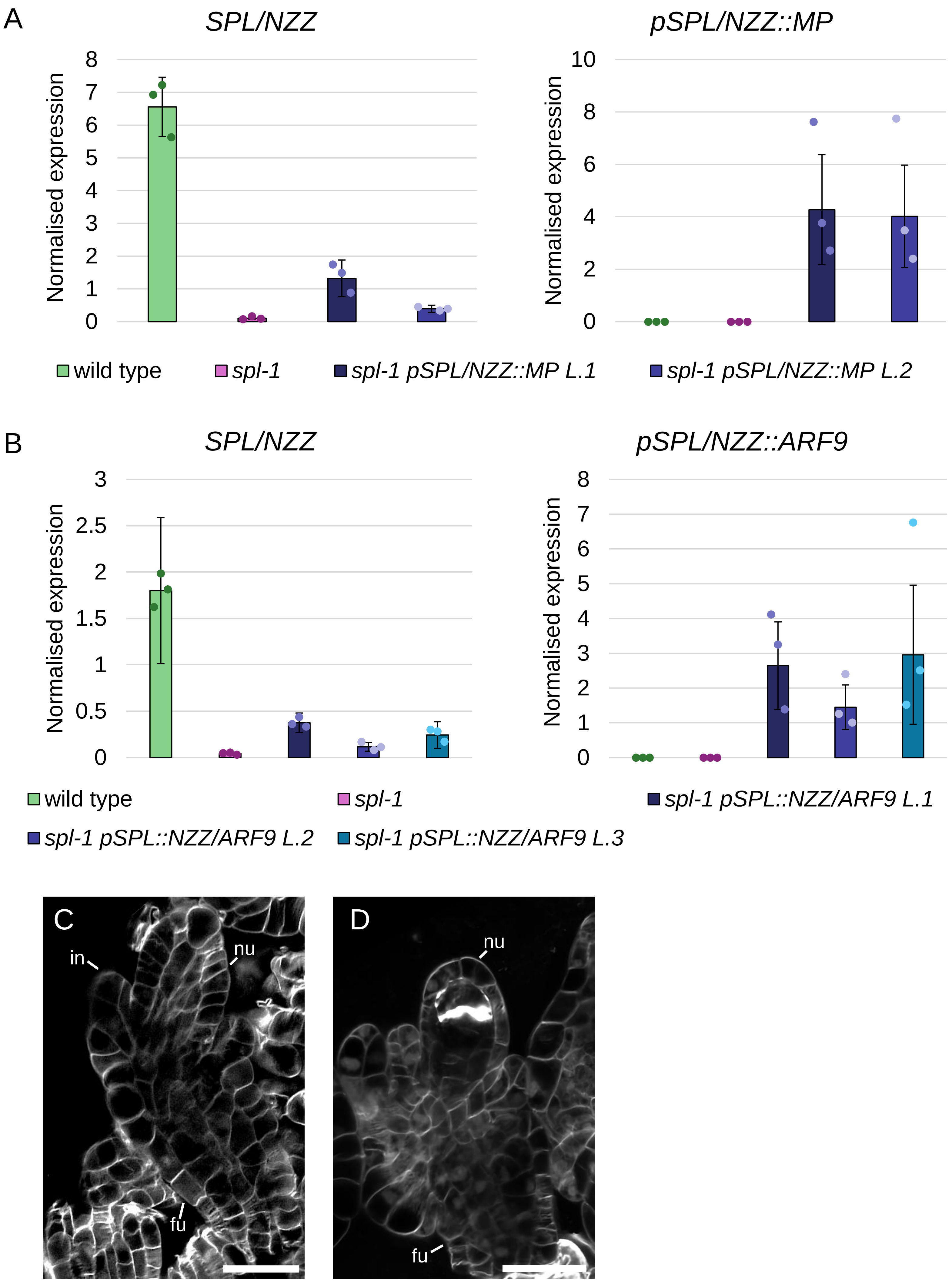

A
SPL/NZZ
Normalised expression
8
7
6
5
4
3
2
1
0
pSPL/NZZ::MP
10
8
6
4
2
0
wild type
spl-1
spl-1 pSPL/NZZ::MP L.1
spl-1 pSPL/NZZ::MP L.2
B
SPL/NZZ
3
2.5
2
1.5
1
0.5
0
pSPL/NZZ::ARF9
wild type
spl-1
spl-1 pSPL::NZZ/ARF9 L.1
spl-1 pSPL::NZZ/ARF9 L.2
spl-1 pSPL::NZZ/ARF9 L.3
C
in
nu
fu
D
nu
fu

Supplementary Figure 14: *pSPL/NZZ::MP* and *pSPL/NZZ::ARF9* expression, *spl-1 pSPL/NZZ::MP* and *spl-1 pSPL/NZZ::ARF9* ovules at stage 2-IV. (**A**) Bar plot showing *SPL/NZZ* and *pSPL/NZZ::MP* normalised expressions in inflorescences from wild type, *spl-1* and two independent *spl-1 pSPL/NZZ::MP* lines. *ACTIN8* was used as housekeeping gene for normalisation. Bars represent the mean ± SEM of the expression, as evaluated from three technical replicates per each sample. Dots within the bars represent individual values for each technical replicate. Source data are provided as a Source Data file. Primers used are listed in Supplementary Data 4. (**B**) Bar plot showing *SPL/NZZ* and *pSPL/NZZ::ARF9* normalised expressions in inflorescences from wild type, *spl-1* and three independent *spl-1 pSPL/NZZ::ARF9* lines. *ACTIN8* was used as housekeeping gene for normalisation. Bars represent the mean ± SEM of the expression, as evaluated from three technical replicates per each sample. Dots within the bars represent individual values for each technical replicate. Source data are provided as a Source Data file. Primers used are listed in Supplementary Data 4. (**C**) In *spl-1 pSPL/NZZ::MP* ovules, the MMC does not proceed into the meiotic process. (**D**) In *spl-1 pSPL/NZZ::ARF9,* the MMC divide meiotically, as indicated by the thick callose deposition associated with the end of meiosis I. Abbreviations: nu= nucellus; fu= funiculus; in= integument. Scale bar = 20 µm.

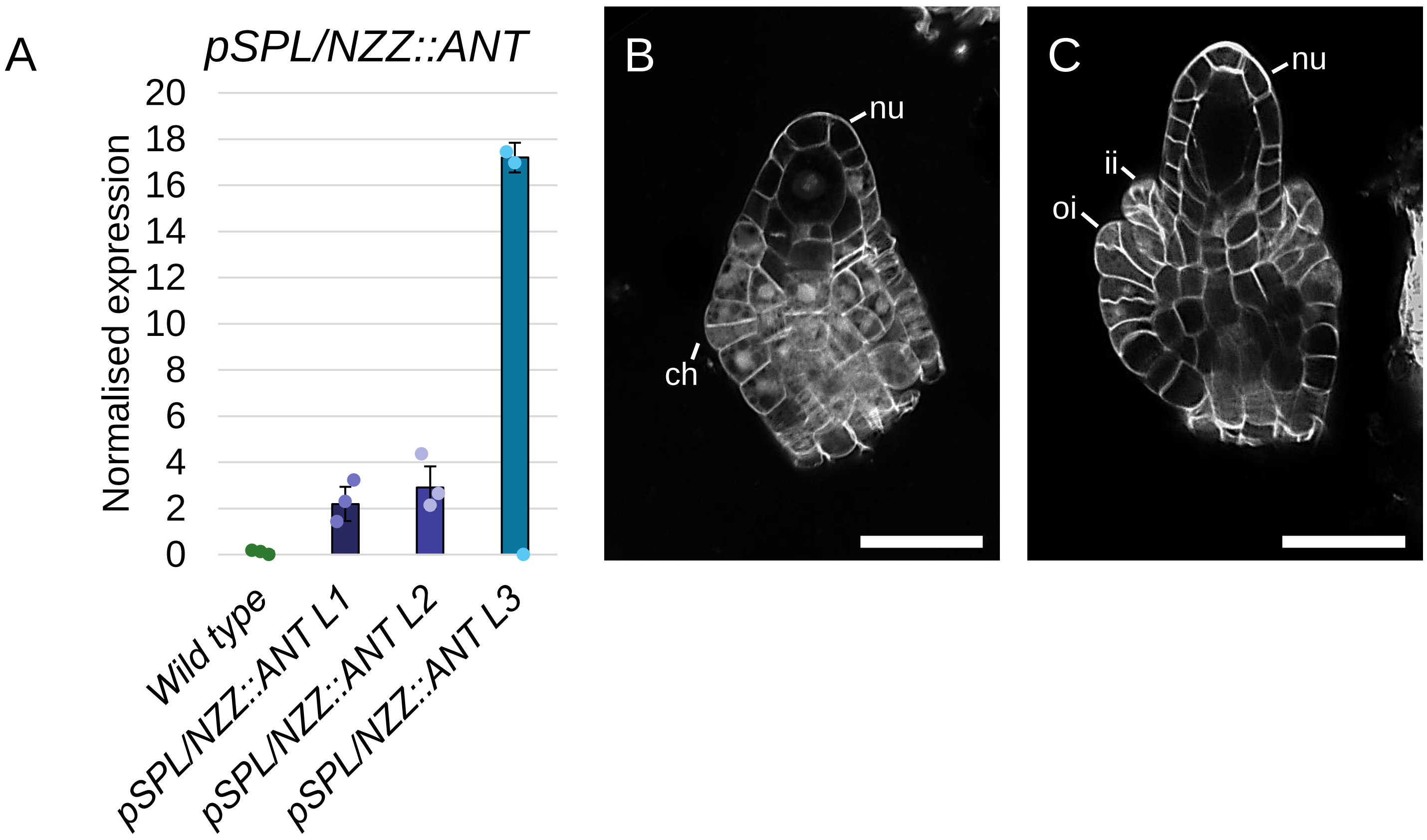

Supplementary Figure 15: Expression of *pSPL/NZZ::ANT* and ovule phenotypes in *pSPL/NZZ::ANT* lines. (**A**) Bar plot showing *pSPL/NZZ::ANT* normalised expressions in inflorescences from wild type and three independent *pSPL/NZZ::ANT* lines. *ACTIN8* was used as housekeeping gene for normalisation. Bars represent the mean ± SEM of the expression, as evaluated from three technical replicates per each sample. Dots within the bars represent individual values for each technical replicate. Source data are provided as a Source Data file. Primers used are listed in Supplementary Data 4. (**B**, **C**) Ovule development in *pSPL/NZZ::ANT* lines. Abbreviations: nu= nucellus; ii= inner integument; oi= outer integument; ch= chalaza; MMC= megaspore mother cell. Scale bar = 20 µm.

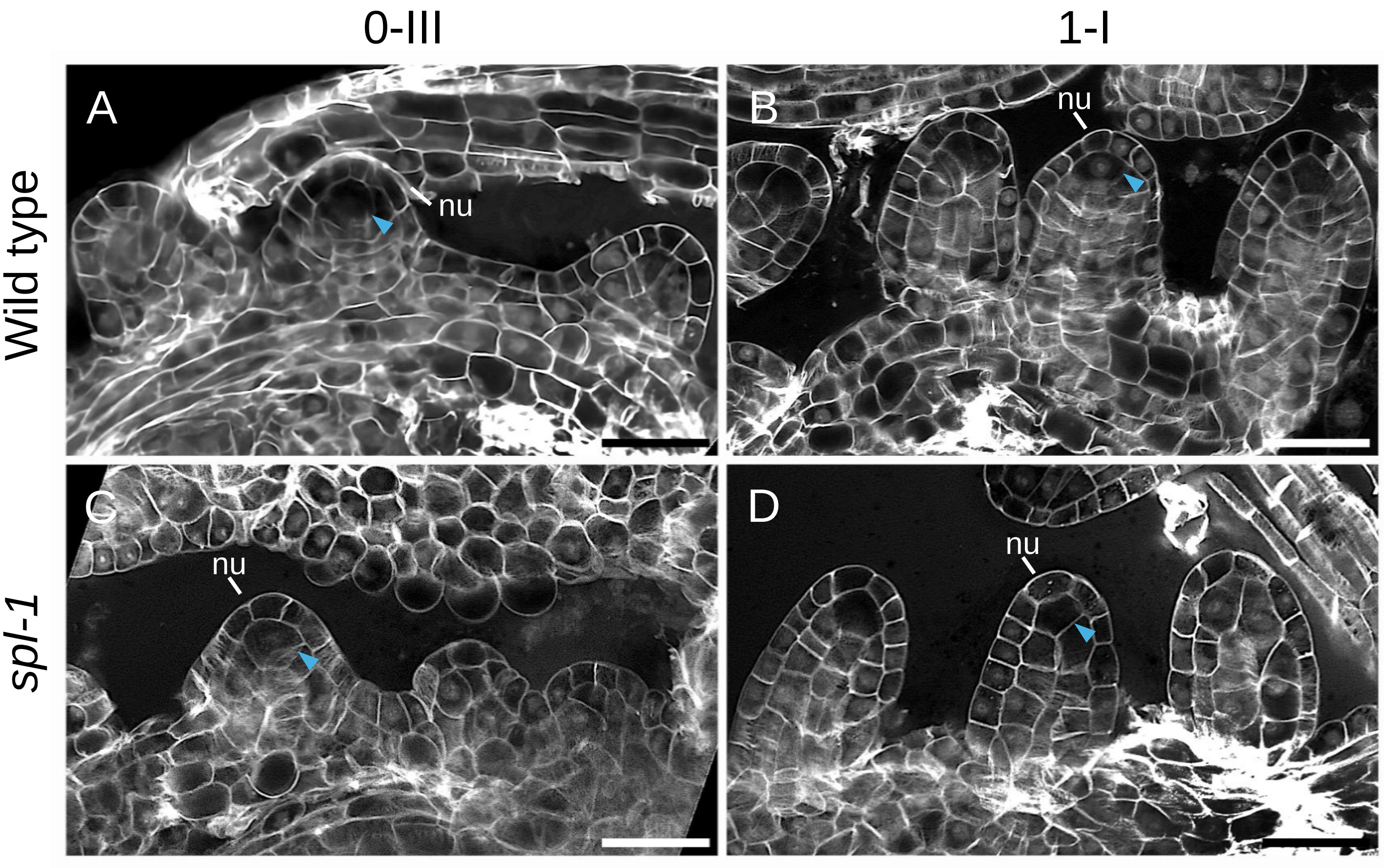

Supplementary Figure 16: Wild-type and *spl-1* ovules at precocious developmental stages. (**A, B**) wild type ovules at stages 0-III (**A**) and 1-I (**B**). (**C**, **D**) *spl-1* ovules at stages 0-III (**C**) and 1-I (**D**). Before MMC specification, wild type and *spl-1* share a similar phenotype, showing the presence of putative MMC precursors in the nucellus. Blue arrowheads indicate the putative MMC precursors. Abbreviations: nu= nucellus. Scale bar = 20 µm.
