# Supplementary material for: SPOROCYTELESS/NOZZLE cooperates with MADS-domain transcription factors to regulate an auxin-dependent network controlling Megaspore-Mother-Cell differentiation
Source: Nat Commun. 2025 Dec 14;17:683. doi: 10.1038/s41467-025-67343-x (PMC12820212; doi:10.1038/s41467-025-67343-x)
Supplement: Supplementary file 2 — Description of Additional Supplementary files [file 41467_2025_67343_MOESM2_ESM.pdf]

Description of Additional Supplementary Files:

Supplementary Data 1: Results of SPL/NZZ Co-IP/MS and SPL/NZZ interactors GO terms enrichment analysis

Supplementary Data 2: Results of SPL/NZZ ChIP-seq, SPL/NZZ targets enrichment against AGRIS and PlantGSAD transcription factor target lists, SPL/NZZ ChIP-seq and SEP3 ChIP-seq peaks overlap, and SPL/NZZ-SEP3 common targets GO terms enrichment analysis

Supplementary Data 3: Differentially expressed genes (DEGs) from the RNA-seq on *spl-1* vs wild type pistils, SPL/NZZ-SEP3 common targets crossed with their expression in the *spl-1* vs wild type RNA-seq, auxin biosynthetic genes expression in the *spl-1* vs wild type RNA-seq and *spl-1* vs wild type RNA-seq DEGs GO terms enrichment analysis

Supplementary Data 4: List of primers used in this study
