# Supplementary material for: SPOROCYTELESS/NOZZLE cooperates with MADS-domain transcription factors to regulate an auxin-dependent network controlling Megaspore-Mother-Cell differentiation
Source: Nat Commun. 2025 Dec 14;17:683. doi: 10.1038/s41467-025-67343-x (PMC12820212; doi:10.1038/s41467-025-67343-x)
Supplement: Supplementary file 7 — Reporting Summary [file 41467_2025_67343_MOESM7_ESM.pdf]

Corresponding author(s): Lucia Colombo

Last updated by author(s): 26-11-2025

Reporting Summary

Nature Portfolio wishes to improve the reproducibility of the work that we publish. This form provides structure for consistency and transparency in reporting. For further information on Nature Portfolio policies, see our Editorial Policies and the Editorial Policy Checklist.

Statistics

For all statistical analyses, confirm that the following items are present in the figure legend, table legend, main text, or Methods section.

n/a	Confirmed	
☐	☒	The exact sample size (*n*) for each experimental group/condition, given as a discrete number and unit of measurement
☐	☒	A statement on whether measurements were taken from distinct samples or whether the same sample was measured repeatedly
☐	☒	The statistical test(s) used AND whether they are one- or two-sided<br>*Only common tests should be described solely by name; describe more complex techniques in the Methods section.*
☒	☐	A description of all covariates tested
☐	☒	A description of any assumptions or corrections, such as tests of normality and adjustment for multiple comparisons
☐	☒	A full description of the statistical parameters including central tendency (e.g. means) or other basic estimates (e.g. regression coefficient) AND variation (e.g. standard deviation) or associated estimates of uncertainty (e.g. confidence intervals)
☐	☒	For null hypothesis testing, the test statistic (e.g. *F*, *t*, *r*) with confidence intervals, effect sizes, degrees of freedom and *P* value noted<br>*Give P values as exact values whenever suitable.*
☒	☐	For Bayesian analysis, information on the choice of priors and Markov chain Monte Carlo settings
☒	☐	For hierarchical and complex designs, identification of the appropriate level for tests and full reporting of outcomes
☒	☐	Estimates of effect sizes (e.g. Cohen's *d*, Pearson's *r*), indicating how they were calculated

Our web collection on statistics for biologists contains articles on many of the points above.

Software and code

Policy information about availability of computer code

Data collection	-For the Co-IP/MS, petides were analysed using the EASY-nLC 1200 (Thermo Fisher ScientificTM) coupled to the Q Exactive Plus mass spectrometer (Thermo Fisher ScientificTM).<br>-For the ChIP-seq experiment, DNA was sequenced with an illumina sequencer<br>- For RNA-seq RNA was sequenced with the Novaseq X, at a depth of 30M reads with a paired-end sequencing.<br>-Confocal imaging was performed using a Nikon AX confocal using the NIS-element software.<br>-FRET-FLIM was performed with a Nikon ECLIPSE Ni-E A1 confocal, using the NIS-element software and the SymPhoTime 64 software

Data analysis	-For the Co-IP/MS, the RAW peptide sequencing data obtained from the Q Exactive Plus were processed by the MaxQuant (version 1.6.14.0) software. After the MaxQuant analysis, the output was analysed in the Perseus (version 1.6.15.0) software.The GO term enrichment analyses were performed with clusterProfiler (R version 4.4.1), with default parameters.<br>-For the ChIP-seq experiment, raw data were trimmed using Trimmomatic 0.39. Mapping was performed using Bowtie 2 (version 2.5). The peak calling was performed with MACS2 (version 2.2.9.1) and merged with BEDTools (version 2.26.0). Peak annotation, using ChIPseeker, and the GO term enrichment analysis were performed on R studio (version 4.4.1)<br>- For the RNA-seq, Raw data were trimmed using Trimmomatic 0.39, Mapping was performed with STAR (version 2.7.10b). Counts were obtained with featureCounts (version 2.0.1) and filtered with HTSFilter. DEGs were obtained using DESeq2. DEGs evaluation and GO terms enrichment analyses were performed on R studio (version 4.4.1)<br>-Confocal images were analysed with the Fiji software<br>-FRET-FLIM data generated by the ymPhoTime 64 software were analysed with the FLIM-Phasor analysis software.<br>-RT-qPCR data were analysed with the Bio-Rad CFX manager (version 3.1)

For manuscripts utilizing custom algorithms or software that are central to the research but not yet described in published literature, software must be made available to editors and reviewers. We strongly encourage code deposition in a community repository (e.g. GitHub). See the Nature Portfolio guidelines for submitting code & software for further information.

Data

Policy information about availability of data

All manuscripts must include a data availability statement. This statement should provide the following information, where applicable:

- Accession codes, unique identifiers, or web links for publicly available datasets
- A description of any restrictions on data availability
- For clinical datasets or third party data, please ensure that the statement adheres to our policy

All data generated in this study are available from the corresponding author upon reasonable request. Source Data underlying Figures 1-6 and Supplementary Figures 5, 7, 9, 10, 11, 14, 15 are provided as a Source Data file. Lists of SPL/NZZ interactors and target genes, as well as differentially expressed genes in spl-1 with respect to the wild type, can be found in the Supplementary Data 1-3. Primers used are listed in Supplementary Data 4.
The mass spectrometry proteomics data have been deposited to the ProteomeXchange Consortium via the PRIDE102 partner repository with the dataset identifier PXD070409 [https://www.ebi.ac.uk/pride/archive/projects/PXD070409].
ChIP-seq raw data have been deposited to the NCBI Sequence Read Archive (SRA) under the accession PRJNA1303074 [https://www.ncbi.nlm.nih.gov/bioproject/?term=PRJNA1303074].
RNA-seq raw data have been deposited to the NCBI Sequence Read Archive (SRA) under the accession PRJNA1365841 [https://www.ncbi.nlm.nih.gov/sra/?term=PRJNA1365841]. Source data are provided with this paper.

Research involving human participants, their data, or biological material

Policy information about studies with human participants or human data. See also policy information about sex, gender (identity/presentation), and sexual orientation and race, ethnicity and racism.

Reporting on sex and gender	*Use the terms sex (biological attribute) and gender (shaped by social and cultural circumstances) carefully in order to avoid confusing both terms. Indicate if findings apply to only one sex or gender; describe whether sex and gender were considered in study design; whether sex and/or gender was determined based on self-reporting or assigned and methods used.*<br>*Provide in the source data disaggregated sex and gender data, where this information has been collected, and if consent has been obtained for sharing of individual-level data; provide overall numbers in this Reporting Summary. Please state if this information has not been collected.*<br>*Report sex- and gender-based analyses where performed, justify reasons for lack of sex- and gender-based analysis.*
Reporting on race, ethnicity, or other socially relevant groupings	*Please specify the socially constructed or socially relevant categorization variable(s) used in your manuscript and explain why they were used. Please note that such variables should not be used as proxies for other socially constructed/relevant variables (for example, race or ethnicity should not be used as a proxy for socioeconomic status).*<br>*Provide clear definitions of the relevant terms used, how they were provided (by the participants/respondents, the researchers, or third parties), and the method(s) used to classify people into the different categories (e.g. self-report, census or administrative data, social media data, etc.)*<br>*Please provide details about how you controlled for confounding variables in your analyses.*
Population characteristics	*Describe the covariate-relevant population characteristics of the human research participants (e.g. age, genotypic information, past and current diagnosis and treatment categories). If you filled out the behavioural & social sciences study design questions and have nothing to add here, write "See above."*
Recruitment	*Describe how participants were recruited. Outline any potential self-selection bias or other biases that may be present and how these are likely to impact results.*
Ethics oversight	*Identify the organization(s) that approved the study protocol.*

Note that full information on the approval of the study protocol must also be provided in the manuscript.

Field-specific reporting

Please select the one below that is the best fit for your research. If you are not sure, read the appropriate sections before making your selection.

☒ Life sciences ☐ Behavioural & social sciences ☐ Ecological, evolutionary & environmental sciences

For a reference copy of the document with all sections, see nature.com/documents/nr-reporting-summary-flat.pdf

Life sciences study design

All studies must disclose on these points even when the disclosure is negative.

Sample size	No sample size calculation was performed. Sample size for each experiment is presented in the figures legend or in the material and methods. Sample size has been determined accordingly to similar studies. Experiment groups were composed by independend observations.
Data exclusions	No data were excluded
Replication	CO-IP/MS, ChIP-seq and RNA-seq are based on the results of three biological replicates respectively. Complementation experiments were performed on independent lines (from a minimum of two to a maximum of 4) .
Randomization	Plant samples, belonging to different plants with the same genotype, were randomly collected for the different analysis performed.
Blinding	No blinding was performed. Key experiments were performed and analysed by at least two researchers.

Reporting for specific materials, systems and methods

We require information from authors about some types of materials, experimental systems and methods used in many studies. Here, indicate whether each material, system or method listed is relevant to your study. If you are not sure if a list item applies to your research, read the appropriate section before selecting a response.

Materials & experimental systems

n/a	Involved in the study	
☐	☒	Antibodies
☒	☐	Eukaryotic cell lines
☒	☐	Palaeontology and archaeology
☒	☐	Animals and other organisms
☒	☐	Clinical data
☒	☐	Dual use research of concern
☐	☒	Plants

Methods

n/a	Involved in the study	
☐	☒	ChIP-seq
☒	☐	Flow cytometry
☒	☐	MRI-based neuroimaging

Antibodies

Antibodies used	-Anti-GFP antibody (abcam, ab290)<br>-µMACS GFP Isolation Kit (Miltenyi Biotec, cat. number: 130-091-288)<br>-Anti-Digoxigenin-AP, Fab fragments (Roche, cat. number: 11093274910)
Validation	-Anti-GFP antibody (abcam, ab290)= Applications:WB, EM, ICC/IF, IHC-Fr, ELISA, IP, IHC-P, IHC-FoFr, IHC-FrFl; Working concentration for ChIP-seq: 1-2 ug per sample. Specificity: GFP antibody is reactive against all variants of Aequorea victoria GFP such as S65T-GFP, RS-GFP, YFP, CFP, RFP and EGFP. (https://www.abcam.com/en-us/products/primary-antibodies/gfp-antibody-ab290)<br>-µMACS GFP Isolation Kit (Miltenyi Biotec, cat. number: 130-091-288)= Application: isolation of GFP fusion proteins from different sources. Working concentration for Co-IP: 50 ul of beads per sample. Specificity: anti-GFP monoclonal antibody, enabling fast and effective magnetic labeling of GFP-tagged fusion proteins (https://www.miltenyibiotec.com/IT-en/products/umacs-and-multimacs-gfp-isolation-kits.html#130-091-288)<br>-Anti-Digoxigenin-AP, Fab fragments (Roche, cat. number: 11093274910)= Application: Anti-Digoxigenin-AP, Fab fragments are useful for the detection of digoxigenin-labeled compounds. Working concentration for in situ hybridisation: 1:700 dilution.<br>Specificity: primary, polyclonal antibodies for the detection of digoxigenin-labeled compounds (https://www.sigmaaldrich.com/IT/en/product/roche/11093274910)

Dual use research of concern

Policy information about dual use research of concern

Hazards

Could the accidental, deliberate or reckless misuse of agents or technologies generated in the work, or the application of information presented in the manuscript, pose a threat to:

No	Yes	
☒	☐	Public health
☒	☐	National security
☒	☐	Crops and/or livestock
☒	☐	Ecosystems
☒	☐	Any other significant area

Experiments of concern

Does the work involve any of these experiments of concern:

No	Yes	
☒	☐	Demonstrate how to render a vaccine ineffective
☒	☐	Confer resistance to therapeutically useful antibiotics or antiviral agents
☒	☐	Enhance the virulence of a pathogen or render a nonpathogen virulent
☒	☐	Increase transmissibility of a pathogen
☒	☐	Alter the host range of a pathogen
☒	☐	Enable evasion of diagnostic/detection modalities
☒	☐	Enable the weaponization of a biological agent or toxin
☒	☐	Any other potentially harmful combination of experiments and agents

Plants

Seed stocks

spl-1 /+ (https://doi.org/10.1105/tpc.112.100164) - pSPL/NZZ::SPL/NZZ:GFP (https://doi.org/10.1242/dev.194274) - AP1:GR/ap1cal (https://doi.org/10.1105/tpc.113.113209) -.shp1, shp2, stk/STK (https://doi.org/10.1105/tpc.107.051797) - ant.4 /+ (DOI 10.1007/s00497-009-0130-3) -pSTK:STK:GFP (https://doi.org/10.1371/journal.pgen.1004856) - pSEP3::SEP3:GFP, pAG::AG:GFP (doi:10.1186/1471-2229-9-5) - spl-1 pPIN1::PIN1:GFP (https://doi.org/10.1105/tpc.112.100164) - pPIN3::PIN3:GFP (https://doi.org/10.1242/dev.041277) - DR5v2 and R2D2 (doi:10.1038/nmeth.3279)

Novel plant genotypes

pSPL/NZZ::SPL/NZZ:GFP pAP1::AP1:GR ap1cal, pSPL/NZZ::MADSas (3 T1 lines), spl-1 pSPL/NZZ::PIN1 (4 T2 lines), pSPL/NZZ::shy2.6 (3 T1 lines), pSPL/NZZ::MP (2 T2 lines), pSPL/NZZ::ARF9 (3 T2 lines) and pSPL/NZZ::ANT (3 T2 lines) were obtained by floral dip technique using the Agrobacterium tumefaciens strain GV3101

Authentication

spl-1 /+; shp1, shp2, stk/STK; ant.4 /+ = PCR-based genotyping for the genetic background
-pSTK:STK:GFP; pSEP3::SEP3:GFP; pAG::AG:GFP = GFP signal imaging
-spl-1 pPIN1::PIN1:GFP; spl-1 pPIN3::PIN3:GFP; spl-1 DR5v2; spl-1 R2D2= PCR-based genotyping for the genetic background and fluorescent signal imaging
-pSPL/NZZ::MADSas; spl-1 pSPL/NZZ::PIN1; pSPL/NZZ::shy2.6; spl-1 pSPL/NZZ::MP; spl-1 pSPL/NZZ::ARF9; pSPL/NZZ::ANT = Selection on medium, PCR-based genotyping for the genetic background and the presence of construct in the genome. RT-qPCR for expression level

ChIP-seq

Data deposition

☒ Confirm that both raw and final processed data have been deposited in a public database such as GEO.

☒ Confirm that you have deposited or provided access to graph files (e.g. BED files) for the called peaks.

Data access links
May remain private before publication.

https://www.ncbi.nlm.nih.gov/bioproject/?term=PRJNA1303074

Files in database submission

SPL/NZZ-GFP IP1
SPL/NZZ-GFP IP2
SPL/NZZ-GFP IP3
SPL/NZZ-GFP input

Genome browser session
(e.g. UCSC)

https://genome-euro.ucsc.edu/cgi-bin/hgTracks?hgS_doOtherUser=submit&hgS_otherUserName=Chiara_Astori&hgS_otherUserSessionName=SPL%2FNZZ%20ChIPseq

Methodology

Replicates	Three biological replicates of immunoprecipitated (IP) samples and one input sample, as reference, were sequenced. The difference between IP and input samples, as well as the similarity among the indipendent IP samples, was assessed by a fingerprint plot.
Sequencing depth	Sequencing depth: 25X<br>Total number of reads:<br>input_1.1: 14958816<br>input_1.2: 14958816<br>IP_1.1: 15692546<br>IP_1.2: 15692546<br>IP_2.1: 17748885<br>IP_2.2: 17748885<br>IP_3.1: 17944528<br>IP_3.2: 17944528<br>Uniquely mapped reads:<br>input_1: 17122900<br>IP_1: 17333984<br>IP_2: 18456196<br>IP_3: 19086798<br>length of the reads: 101 bp<br>paired-end sequencing
Antibodies	Anti-GFP antibody (abcam, ab290)
Peak calling parameters	read mapping:<br>bowtie2 -p 10 Bowtie2_indexed -1 TrimmedData/${name}_1P.tr.fq.gz -2 TrimmedData/${name}_2P.tr.fq.gz<br>Bowtie2_indexed is the Arabidopsis TAIR10 genome indexed by bowtie2-build.<br>Peak calling:<br>macs2 callpeak -t L150459_Track-195043.sort.rmdup.M30.bam -c L150457_Track-195041.sort.rmdup.M30.bam -f BAMPE -g 118459858 -n 43vs41 --outdir MACS2/<br>macs2 callpeak -t L150459_Track-195043.sort.rmdup.M30.bam -c L150457_Track-195041.sort.rmdup.M30.bam -f BAMPE -g 118459858 -n 44vs41 --outdir MACS2/<br>macs2 callpeak -t L150461_Track-195045.sort.rmdup.M30.bam -c L150457_Track-195041.sort.rmdup.M30.bam -f BAMPE -g 118459858 -n 45vs41 --outdir MACS2/<br>L150459_Track-195043.sort.rmdup.M30.bam, L150459_Track-195043.sort.rmdup.M30.bam and L150461_Track-195045.sort.rmdup.M30.bam are the immunoprecipitated samples, whereas the L150457_Track-195041.sort.rmdup.M30.bam is the input sample.
Data quality	Reads were trimmed by trimmomatic to remove adaptors and low quality reads (AVGQUAL:20). After mapping, duplicated reads were discarded, as well as reads with a mapping quality lower that 30 (-q 30). During peak calling, MACS2 automatically discards the peaks with a q-value higher that 5.00e-02. Peaks were merged by BEDTools, and we kept for further analysis just the peaks that were present in at least two out of three replicates.<br>This approach retrived a list of 203 peaks with FDR lower than 5% . The lower fold enrichment measured was around 1.6, whereas the highest one was 31. peaks with a fold enrichment above 5 are 67.
Software	Raw data were trimmed using Trimmomatic 0.39. Mapping was performed using Bowtie 2 (version 2.5). Filtering was performed with SAMtools (version 1.7). The peak calling was performed with MACS2 (version 2.2.9.1) and merged with BEDTools (version 2.26.0). Peak annotation, using ChIPseeker, and the GO term enrichment analysis were performed on R studio (version 4.4.1)
